# Key metabolism pathways and regulatory mechanisms of high polysaccharide yielding in *Hericium erinaceus*

**DOI:** 10.1186/s12864-021-07480-x

**Published:** 2021-03-06

**Authors:** Ming Gong, Henan Zhang, Di Wu, Zhong Zhang, Jinsong Zhang, Dapeng Bao, Yan Yang

**Affiliations:** Institute of Edible Fungi, Shanghai Academy of Agricultural Sciences, National Engineering Research Center of Edible Fungi, Key Laboratory of Edible Fungi Resources and Utilization (South), Ministry of Agriculture, the People’s Republic of China, No.1000, Jinqi Road, Shanghai, 201403 China

**Keywords:** *Hericium erinaceus*, ARTP mutagenesis, High polysaccharide yield, Carbohydrate metabolism, RAS-cAMP-PKA pathway

## Abstract

**Background:**

*Hericium erinaceus*, a rare edible and medicine fungus, is widely used in the food and medical field. Polysaccharides from *H. erinaceus* are the main bioactive compound that exert high bioactive value in the medical and healthcare industries.

**Results:**

The genome of *H. erinaceus* original strain HEA was reported 38.16 Mb, encoding 9780 predicted genes by single-molecule, real-time sequencing technology. The phylogenomic analysis showed that *H. erinaceus* had the closest evolutionary affinity with *Dentipellis sp*. The polysaccharide content in the fermented mycelia of mutated strains HEB and HEC, which obtained by ARTP mutagenesis in our previous study, was improved by 23.25 and 47.45%, and a new β-glucan fraction with molecular weight 1.056 × 10^6^ Da was produced in HEC. Integrative analysis of transcriptome and proteomics showed the upregulation of the carbohydrate metabolism pathway modules in HEB and HEC might lead to the increased production of glucose-6P and promote the repeating units synthesis of polysaccharides. qPCR and PRM analysis confirmed that most of the co-enriched and differentially co-expressed genes involved in carbohydrate metabolism shared a similar expression trend with the transcriptome and proteome data in HEB and HEC. Heatmap analysis showed a noticeably decreased protein expression profile of the RAS-cAMP-PKA pathway in HEC with a highly increased 47.45% of polysaccharide content. The S phase progression blocking experiment further verified that the RAS-cAMP-PKA pathway’s dysfunction might promote high polysaccharide and β-glucan production in the mutant strain HEC.

**Conclusions:**

The study revealed the primary mechanism of the increased polysaccharide synthesis induced by ARTP mutagenesis and explored the essential genes and pathways of polysaccharide synthesis.

**Supplementary Information:**

The online version contains supplementary material available at 10.1186/s12864-021-07480-x.

## Background

*Hericium erinaceus* is a famous precious food and medicine fungus in China, and it has become a valuable resource for the functional food and medicine industry [1]. Polysaccharides from *H. erinaceus* are the main bioactive compound, which exerts many biological activities, including improving immunity, anti-cancer, blood lipids lowering, anti-oxidation, gastro-protective, hypoglycemic activity, and anti-aging [2, 3]. In general, polysaccharides of *H. erinaceus* are mainly obtained from the fruiting body and liquid submerged fermentation mycelium, which yield will be affected by strain, culture conditions, and environmental regulation [4–6]. Further, it is an effective way to improve the quality of fruiting body and mycelia of *H. erinaceus* by breeding strains with high polysaccharide yield. In our previous study, two mutant strains (HEB and HEC) of *H. erinaceus* with higher polysaccharide production were bred by atmospheric pressure room temperature plasma (ARTP) mutagenesis, and the polysaccharide production in liquid fermentation mycelium and fruiting bodies were both significantly enhanced compared with the original strain [6]. However, the reason and mechanism for the high polysaccharide yield from *H. erinaceus* mutant strain need to be further identified.

In recent years, with the development of structural analysis and functional activity evaluation of polysaccharides from mushroom such as *Ganoderma lucidum* [7], *H. erinaceus* [8], *Cordyceps militaris* [9], *Grifola frondosa* [10], coupled with the gradually clear genetic background of edible fungi, more and more attention has been paid to the biosynthesis process of polysaccharides from edible fungi, including the key enzymes and genes. For example, the production of *G. lucidum* polysaccharide was improved in liquid submerged fermentation mycelium by regulating the Vitreoscilla hemoglobin gene-mediated enzymes participating in polysaccharide biosynthesis, including UDP glucose pyrophosphorylase (UGP), β-1,3-glucan synthase (GLS), and α-phosphoglucomutase (PGM) [11]. Peng et al. reported that the ratio of the monosaccharide composition of *G. lucidum* exopolysaccharide was associated with the activities of PGM, phosphomannose isomerase (PMI), UGP, and phosphoglucose isomerase (PGI), respectively [12]. Another study found that the production and monosaccharide composition of *C. militaris* polysaccharides were manipulated by altering the transcription level of PGM, UGP, and PGI genes [13]. A putative mushroom polysaccharide biosynthetic pathway was proposed based on identifying intermediate compounds, synthesis-related enzymes and key genes disclosure in previous publications [14], which provides a reference for studying biosynthesis pathways in mushroom polysaccharides. So far, there are few reports related to the synthesis of intracellular polysaccharides of *H. erinaceus*, the key genes and the efficient biosynthesis pathway of *H. erinaceus* polysaccharide still need to be further explored.

With the advent of the post-genomic era, the biosynthesis and regulation of intracellular polysaccharides of edible fungi can be revealed through genomics, transcriptomics, and proteomics analysis, which will lay a foundation for high yield of active polysaccharides and the development of edible fungi products [15]. For instance, Tan et al. confirmed that a total of 48 differential expressed genes were related to polysaccharide synthesis and carbohydrate metabolism in *G. lucidum* by high-throughput RNA-sequencing (RNA-seq) [15]. Simultaneously, many genes of *H. erinaceus* involved in polysaccharide biosynthesis were identified using RNA-seq, and these transcripts encoded the key-enzymes related to polysaccharide biosynthesis, including PGM, UGP, and PGI [16]. However, few studies have reported the critical regulatory genes or key enzymes in the biosynthesis pathway of *H. erinaceus* polysaccharide. Intriguingly, recently several studies utilized integration of multi-omics strategy to reveal the biosynthesis of bioactive secondary metabolites (such as terpenoid, polyketide, sterol and triterpene saponin) of *H. erinaceus* [17], *Phellinus linteus* [18], *Wolfiporia cocos* [19], and *Termitomyces albuminosus* [20]. Moreover, Wang et al. found that a total of 47 key enzymes related to the biosynthesis of secondary metabolites and polysaccharides of *G. lucidum* were succinylated through proteomics and bioinformatics analysis, indicating that lysine succinylation exhibits an important role in the biosynthesis of the active compounds in *G. lucidum* [21]. Chen et al. demonstrated that diverse enzymes and cytochrome P450 involved in the secondary metabolite biosynthesis of *H. erinaceus* by genomic and transcriptomic analysis [22]. The above results indicated that multi-omics analysis might also be a possible method to reveal the intracellular polysaccharide biosynthesis pathway of *H. erinaceus*.

In the present study, the high-yielding polysaccharide strains HEB and HEC of *H. erinaceus* obtained by ARTP mutagenesis and the original strain HEA were used as research materials. Multi-omics analysis based on polysaccharide structure difference was employed to predict the biosynthetic pathway and functional genes associated with high intracellular polysaccharide production of *H. erinaceus*. The effect of a repressor of the regulatory pathway on polysaccharides synthesis will further validate the multi-omics analysis results. This study would provide candidate key genes and pathways for improving the intracellular polysaccharides of *H. erinaceus*, and laid a foundation for rational regulation of intracellular polysaccharide synthesis and the cultivation of high-quality resources of *H. erinaceus*.

## Results and discussion

### Culturing of *H. erinaceus* with high intracellular polysaccharide production

**Fig. 1:**
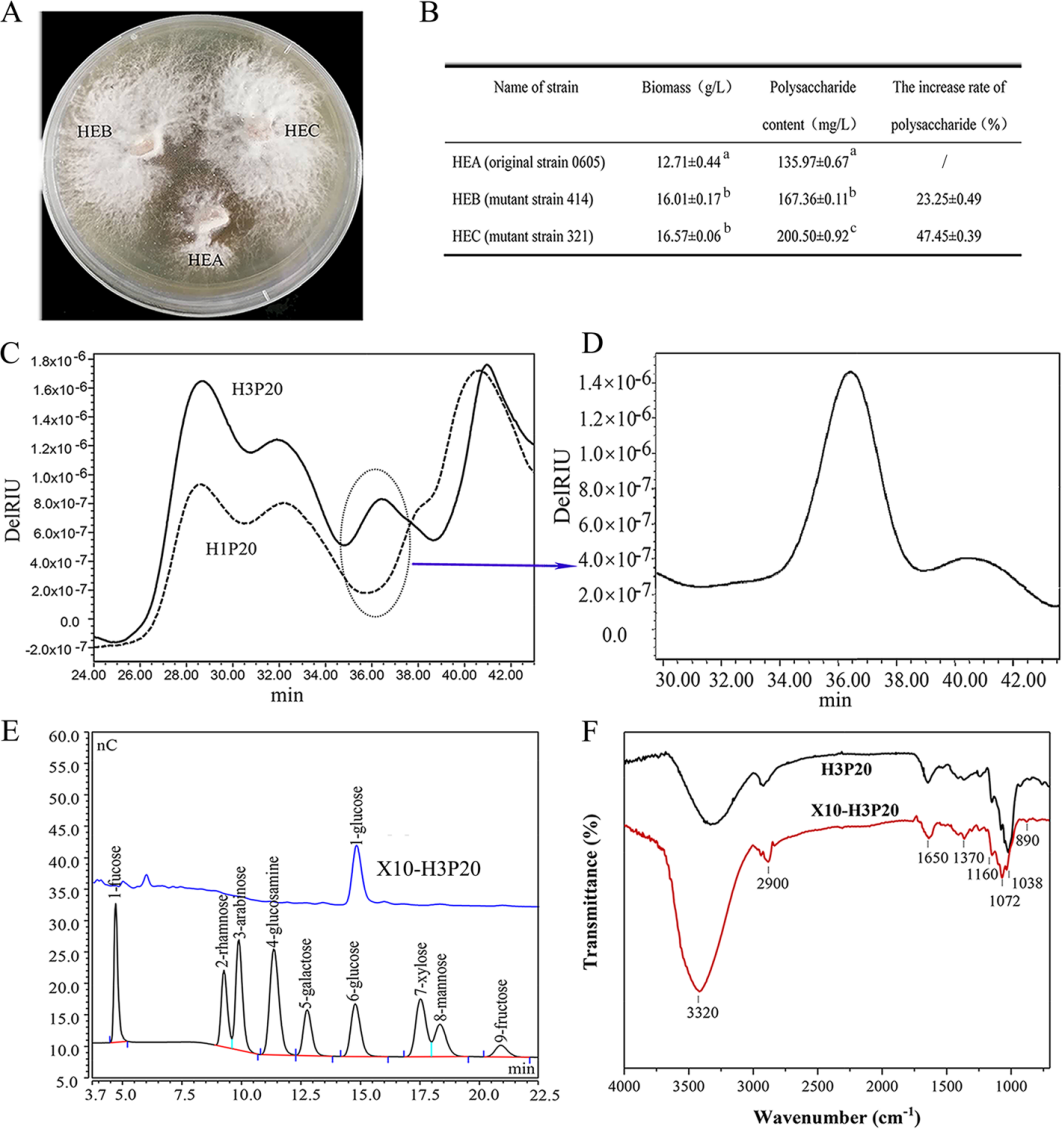
Comparison of biomass, content, structural characteristics of polysaccharide between HEA and the mutated strains. **a** The mutagenic strains HEB and HEC from HEA identified by an antagonism test. **b** The biomass and polysaccharide content of *H. erinaceus* mycelia fermented by bred strains. Different letters indicated *P* < 0.01. **c** HPSEC-MALLS-RI chromatograms of 20% ethanol precipitated polysaccharides from *H. erinaceus* HEA and HEC. **d** The molecular weight distribution of differential polysaccharide X10-H3P20. **e** HPAEC of the monosaccharide composition of X10-H3P20. **f** Infrared spectrogram of H3P20 and X10-H3P20. Note:H1P20 represents the original strain HEA (0605) 20% ethanol precipitated polysaccharide fractions; H3P20 represents the ARTP mutagenic strain HEC (321) 20% ethanol precipitated polysaccharide fractions; X10-H3P20 represents the differential polysaccharide purified from H3P20

In our previous study, the mutant strains of *H. erinaceus* HEB and HEC with high intracellular polysaccharide production were obtained by ARTP mutagenesis [22]. There was an apparent antagonistic reaction between the original strain and the mutants (Fig. 1a). The biomass of liquid fermentation of strain HEB and HEC was higher than that of the original strain HEA, with an increased rate of 25.96 and 30.37%, respectively. The polysaccharide content in the fermented mycelia of the mutant strains HEB and HEC was increased by 23.25 and 47.45% than HEA (Fig. 1b). Statistical analysis showed that the polysaccharide content of the HEC and HEB was significantly different from HEA, which further indicated that ARTP mutagenesis changed polysaccharide production.

The 20% ethanol precipitated polysaccharide fraction of the mutants had higher molecular weight than that of the original strain, and the proportion of glucose and mannose in the polysaccharide components was increased significantly in the mutants than the original strain [22]. An obvious different polysaccharide fraction X10-H3P20 between HEA and HEC was revealed by high-performance size-exclusion chromatography equipped with multiple angle laser light scattering and refractive index detectors (HPSEC-MALLSRI), as shown in Fig. 1c. The molecular weight of this purified polysaccharide X10-H3P20 was about 1.056 × 10^6^ Da (Fig. 1d), and the monosaccharide composition was mainly composed of glucose with a ratio of 92% (Fig. 1e) and meanwhile with a β-configuration glycosidic bonds showed by IR spectrum (Fig. 1f). Our previous study showed that the immunological activity of mutant strain HEC in vitro was better than that of the original strain HEA [22]. This new β-glucan fraction with large molecular weight produced in HEC indicated that ARTP mutagenesis resulted in the synthesis of macromolecule dextran, which enriched the types of polysaccharide compounds, as well as provided more options for screening biological activity.

### Genome sequencing and general features

**Fig. 2:**
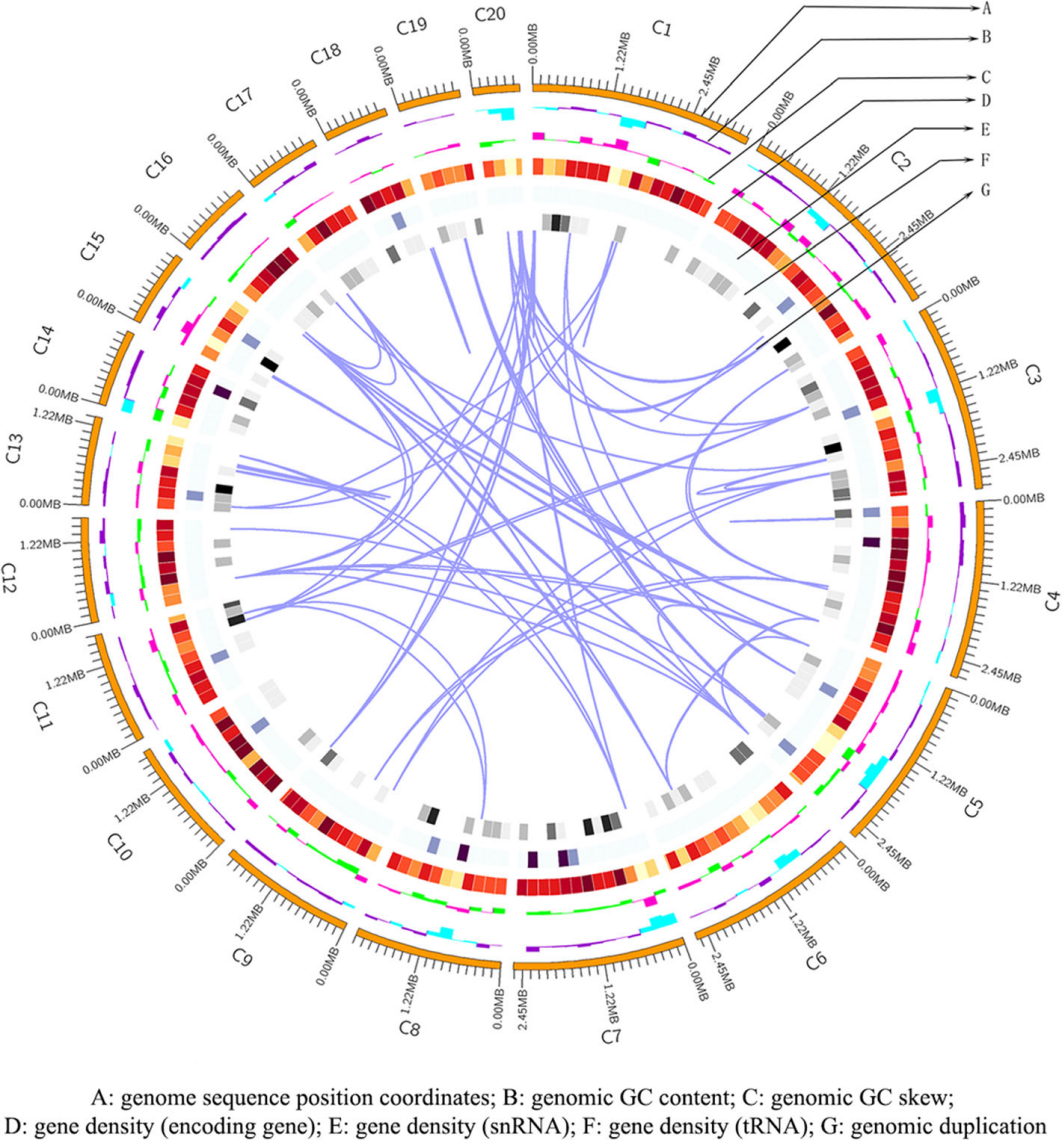
An ideogram showing the genomic features of *H. erinaceus*. **a** Positional coordinates of the genome sequence. **b** GC content was calculated as the percentage of G + C in 200-kb non-overlapping windows. Higher peaks indicate a greater difference with average GC content. **c** GC Skew value was calculated as the percentage of G-C / G + C in 200-kb non-overlapping windows. Higher peaks indicate a greater difference with the average GC Skew value. **d**, **e**, **f** Gene density was represented as the number of coding genes, snRNA and tRNA in 200-kb non-overlapping windows, respectively. The intensity of the color correlates with gene density. **g** Genome duplication

The *H. erinaceus* genome sequences were assembled using SMRT Link v5.0.1 and then evaluated through aligning reads to the assembled sequence to get the final assembly result. The 20 scaffolds were assembled with an N50 of 258.72 kb and a total genome size of 38.16 Mb (Fig. 2 and Table 1). Prediction of the assembled genome sequence generated 9780 gene models. The average length of coding genes was 1355 bp, and the ratio of the total length of the coding region to the whole genome was 34.74%. The average size of exons was 235 bp, and the average size of introns was 70 bp. The 7137 genes encoded proteins with homologous sequences in the NCBI nr protein databases, and 6854 genes were mappable through the KEGG pathway database [23] (Table 1). Functional annotation analysis showed the general features, such as 5611 conserved protein domains (containing 333 CLAN), 2831 proteins involved in different pathways, 5611 proteins divided into different GO terms, and 1822 proteins assigned to different KOG classes in Table 1.

**Table 1:** General features of the *H. erinaceus* genome

General features	number	General features	number
Size of assembled genome (Mb)	38.16	Pfam (genes)	5611
N50_Length (Kb)	258.72	Pfam (CLAN)	333
GC content (%)	53	SwissProt	2267
Length of classified repeats (%)	5	KEGG alignment	6854
Number of predicted gene models	9780	GO assignment	5611
Average exon size (bp)	235	KOG assignment	1822
Average intron size (bp)	70	TCDB	277
Average gene length (bp)	1355	DFVF	360
% of Genome (Genes)	34.74	PHI	406
% of Genome (internal)	65.26	P450	95
Number of tRNA genes	204	Secretory Protein	397
Number of Contigs	20	CAZy	259
NR alignment	7137	Secondary metabolism clusters	19

**Fig. 3:**
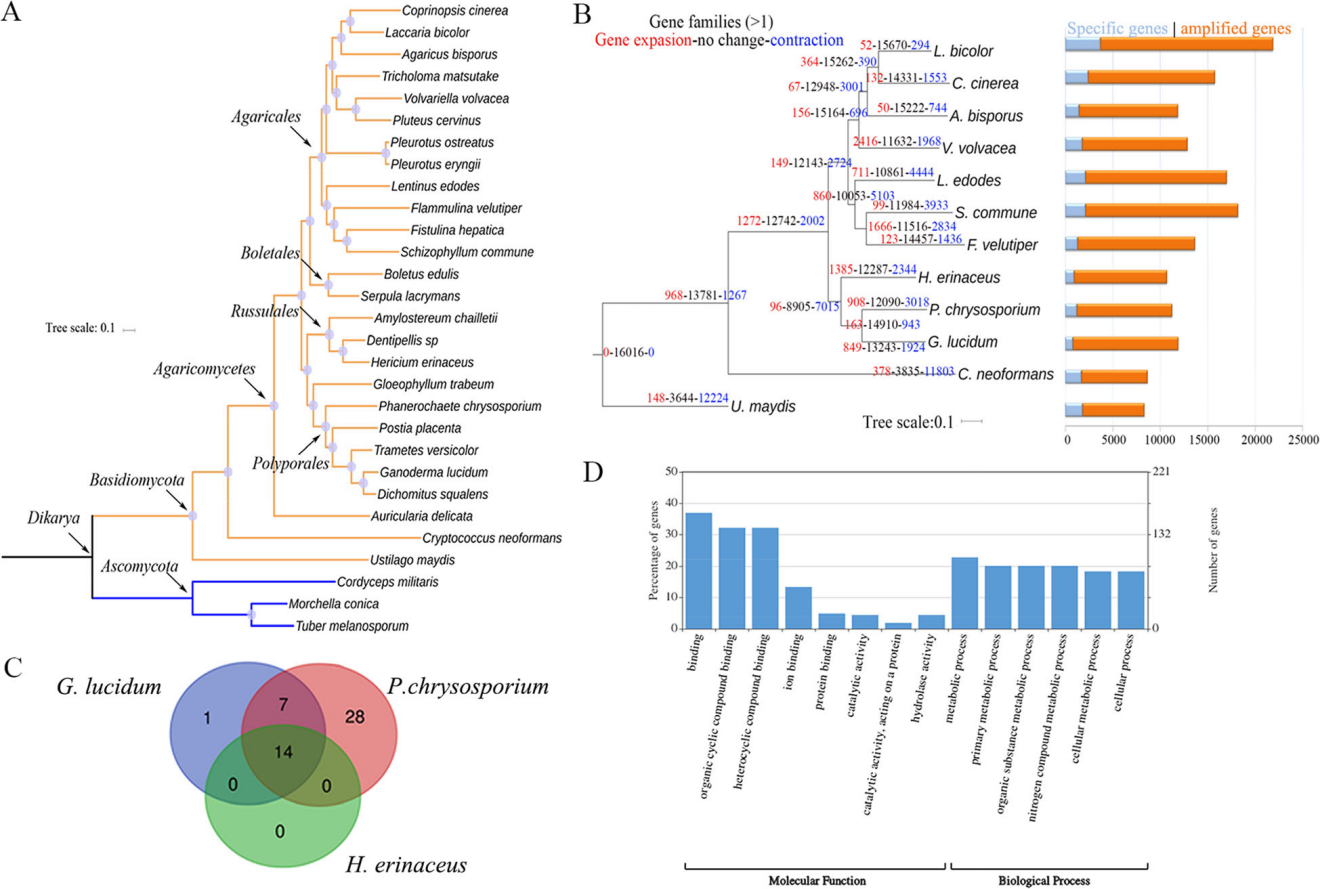
Comparative genomics analysis of *H. erinaceus*. **a** Phylogenomic analysis of *H. erinaceus*. The Maximum-likelihood tree was constructed based upon the concatenated sequences consisting of the single-copy orthologous sequences. **b** Analysis of changes in size and number of gene families in representative basidiomycetes. **c** Comparative analysis of GO annotation for gene families with big size*.* The number of gene families with big size in each species is > = 10, and three times more than those in other species. **d** WEGO analysis of the enriched genes (> = 10) of *H. erinaceus*

The phylogenomic analysis showed that *H. erinaceus* had the closest evolutionary affinity with *Dentipellis sp* (Fig. 3a). The two species were located at the cluster of Russulales and shared a common ancestor with Polyporales. Analysis of gene family size showed that the net value was − 6919 at the node leading to Russulales and Polyporales, indicating a large number of gene loss during the evolution of Russulales and Polyporales (Fig. 3b). Venn analysis of gene families with big size showed no specific GO annotation of *H. erinaceus* compared to those in two species in the same cluster based on the phylogenomic tree (Fig. 3c). WEGO analysis of the enriched genes (> = 10) of *H. erinaceus* showed that metabolic process, primary metabolic process, and other types of metabolic processes belong to the enriched biological process. Binding (GO:0005488) and different kinds of binding (GO:0097159, GO:1901363, GO:0043167, GO:0005515) occupied the most enriched terms of molecular function (Fig. 3d).

### Comparative transcriptome analysis of *H. erinaceus*

**Fig. 4:**
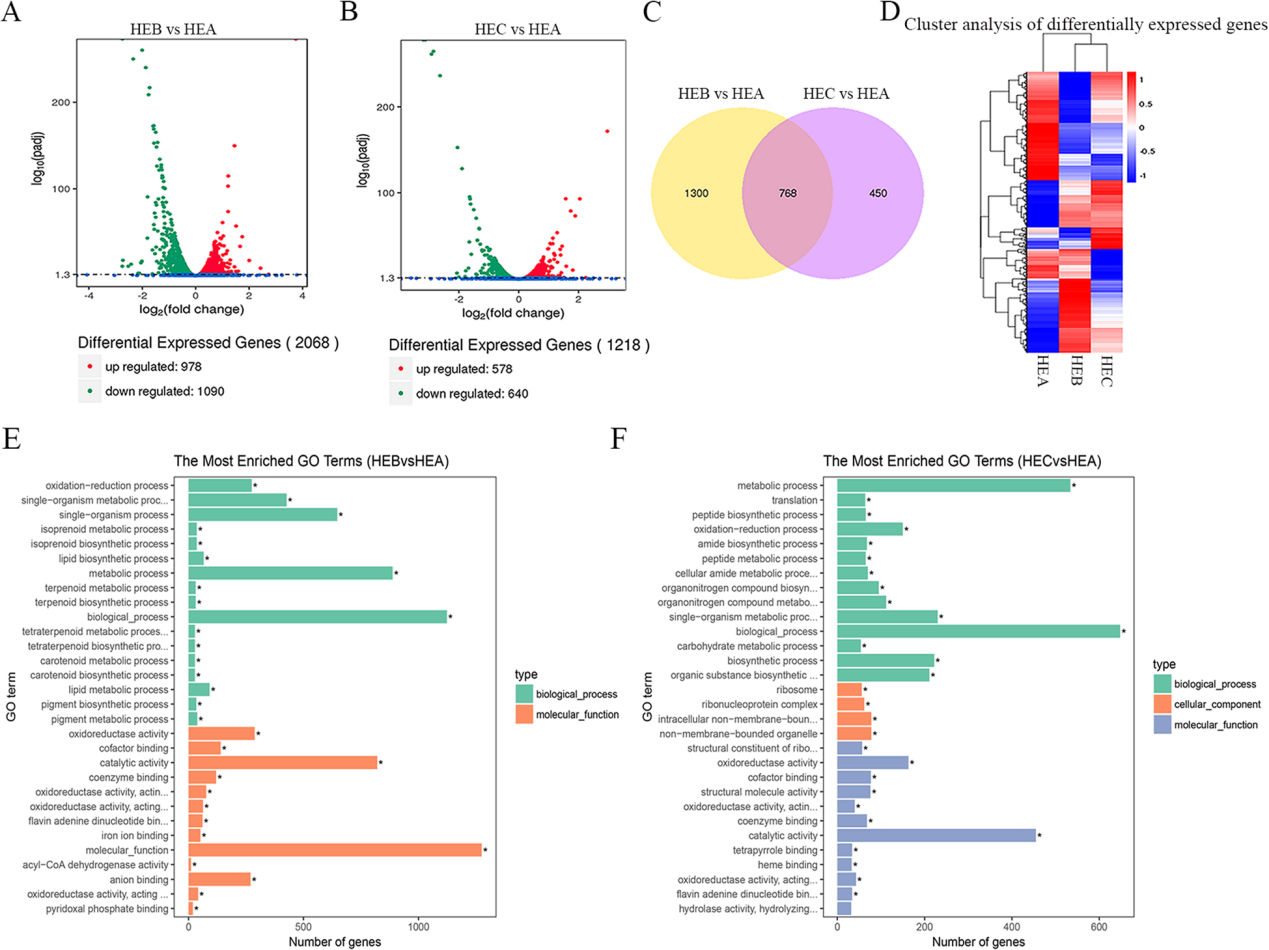
Transcriptome analysis of *H. erinaceus*. **a** Volcano plot analysis of DEGs in HEB_vs_HEA. **b** Volcano plot analysis of DEGs in HEC_vs_HEA. **c** Venn analysis of DEGs. **d** Cluster analysis of DEGs. The blue indicates downregulated mRNAs; the red indicates upregulated mRNAs. **e** GO enrichment analysis of the DEGs in HEB_vs_HEA. **f** GO enrichment analysis of the DEGs in HEC_vs_HEA

The transcriptome analysis of *H. erinaceus* was carried out through the steps of RNA sample extraction, detection, library construction, and sequencing. Results showed 2068 differentially expressed genes (DEGs) in HEB_vs_HEA and 1218 DEGs in HEC_vs_HEA (Fig. 4a and b). Venn analysis showed 768 differentially co-expressed genes among the comparison groups of HEB_vs_HEA and HEC_vs_HEA (Fig. 4c). Heatmap analysis of DEGs showed that HEB and HEC were clustered together (Fig. 4d). GO enrichment analysis of DEGs showed that HEB and HEC had the similar most enriched GO entries, such as biological process, metabolic process, single-organism metabolic process (Fig. 4e and f). The GO entries showed that oxidoreductase, catalytic activity were both enriched in HEB_vs_HEA and HEC_vs_HEA (Fig. 4e, and f), which might be closely related to the synthesis of polysaccharides according to the previous reports [24]. The GO term of cellular components was enriched in HEC_vs_HEA, such as ribosome, ribonucleoprotein complex (Fig. 4f).

KEGG pathway enrichment using KOBAS (2.0) showed that the significantly upregulated genes in HEB_vs_HEA were enriched in the starch and sucrose metabolism, carbon metabolism, and pyruvate metabolism (Additional file 1). The significantly upregulated genes in HEC_vs_HEA were enriched in glycerolipid metabolism, starch and sucrose metabolism, carbon metabolism, pyruvate metabolism, glycolysis/gluconeogenesis (Additional file 2). The significantly downregulated genes in HEB_vs_HEA or HEC_vs_HEA were both enriched in the ribosome (Additional files 3 and 4).

Functional enrichment analysis based on the STRING database showed that the significantly co-upregulated genes in HEB_vs_HEA and HEC_vs_HEA were enriched in metabolic pathways, carbon metabolism, pentose and glucuronate interconversions, pyruvate metabolism (Additional file 5A and B). The significantly co-downregulated genes in HEB_vs_HEA and HEC_vs_HEA were enriched in the ribosomal pathway (Additional file 5 C and D).

These results indicated that the upregulated pathways presented in mutant strains HEB and HEC were involved in carbohydrate metabolism, and the downregulated pathways were strictly associated with protein translation.

### Comparative proteomics analysis of *H. erinaceus*

**Fig. 5:**
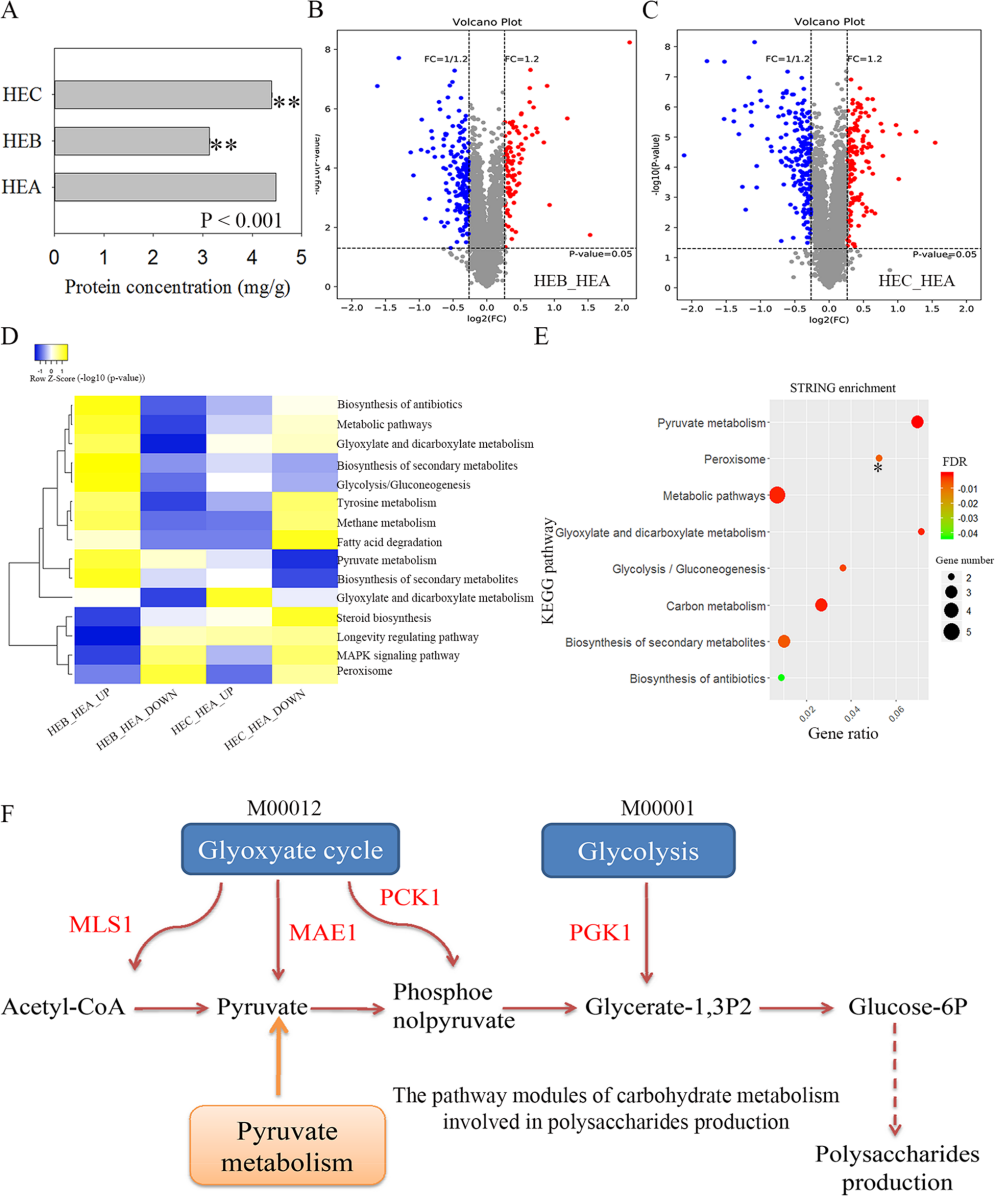
Comparative proteomics analysis of *H. erinaceus*. **a** Protein concentration determination using the BCA method. **b** Volcano plot analysis of DEPs of HEB_vs_HEA. **c** Volcano plot analysis of DEPs in HEC_vs_HEA. **d** The co-enriched KEGG pathways in HEB_vs_HEA and HEC_vs_HEA. The co-enriched KEGG pathways were obtained from the Venn analysis of enriched KEGG pathways in HEB_vs_HEA and HEC_vs_HEA in Additional files 10, 11, 12 and 13. The DEPs were used for KEGG pathway enrichment. Yellow indicates strong enrichment, and blue indicates weak enrichment. **e** STRING network analysis of enriched and differentially co-expressed proteins. These proteins were obtained from the Venn analysis of the DEPs in the enriched pathways in HEB_vs_HEA and HEC_vs_HEA. These circles represent the enrichment results using the co-upregulated proteins, and * represents the enrichment results using the co-downregulated proteins. **f** The pathway modules of carbohydrate metabolism leading to the production of glucose-6P. Red represents the apparent upregulation of MAE1(A6232), MLS1(A4695), PCK1(A5260) in the glyoxylate cycle modules (M00012), and PGK1 (A8906) in the glycolysis module (M00001)

Results of protein concentration determination using the Bicinchoninic Acid (BCA) method confirmed that protein concentration decreased in HEB and HEC compared to HEA (Fig. 5a), partially in agreement with the downregulated mRNA expression in the ribosomal pathway in HEB and HEC (Additional files 3 and 4, Additional file 5 C and D). The principal component analysis showed that HEA, HEB, and HEC had excellent repeatability and discrimination (Additional file 6A). According to the standard Score Sequest HT > 0, unique peptide ≥1, and the blank value was removed, 4555 trusted proteins were screened (Additional file 7). Results of differentially expressed proteins (DEPs) screening (fold change ≥1.2, *p*-value < 0.05) identified 343 DEPs in HEB_vs_HEA and 266 in HEC_vs_HEA (Fig. 5b and c). The details of the DEPs could be found in Additional files 8 and 9. Venn analysis showed that 122 differentially co-expressed proteins in HEB_vs_HEA and HEC_vs_HEA (Additional file 6B).

Heatmap analysis showed the strong enrichment pathways from the significantly upregulated proteins in HEB_vs_HEA and HEC_vs_HEA, such as pyruvate metabolism, glyoxylate and dicarboxylate metabolism, and glycolytic/gluconeogenesis (Fig. 5d). Several strong co-enriched pathways were clustered from the significantly down-regulated proteins in HEB_vs_HEA and HEC_vs_HEA, such as longevity regulation, peroxisome, and MAPK signaling pathway (Fig. 5d). The details about all the enriched KEGG pathways in HEB_vs_HEA or HEC_vs_HEA could be found in Additional files 10, 11, 12 and 13. The 18 co-upregulated proteins from the enriched pathways in Additional files 10, 11, 12 and 13 were enriched in the pathways, such as carbon metabolism, glycolysis/gluconeogenesis, pyruvate metabolism (Fig. 5e), which conformed to the transcriptome analysis results (Additional file 1 and S2). The 22 co-downregulated proteins from the enriched pathways in Additional files 10, 11, 12 and 13 were enriched in the pathways of peroxisome (Fig. 5e).

The KEGG mapping of the pathway modules of carbohydrate metabolism in carbon metabolism in HEB_vs_HEA or HEC_vs_HEA showed the apparent upregulation of MLS1 (A4695), MAE1 (A6232), PCK1 (A5260) in the glyoxylate cycle modules (M00012) and the apparent upregulation of PGK1 (A8906) in the glycolysis module (M00001) (Fig. 5f, Additional file 14). Together with the enrichment of carbon metabolism pathway using the significantly upregulated expressed mRNA in HEB_vs_HEA (Additional file 1) or HEC_vs_HEA (Additional file 2), these observations confirmed the upregulation activities of the pathway modules of carbohydrate metabolism. The two modules (M00012 and M00001) were linked together, leading to the production of glucose-6P, which meant that the upregulated activity of the two modules could promote the production of glucose-6P (Fig. 5f) and further provided the intermediates for polysaccharide synthesis.

### Multi-omics analysis of the hypothesized mushroom polysaccharides production biosynthetic pathways

**Fig. 6:**
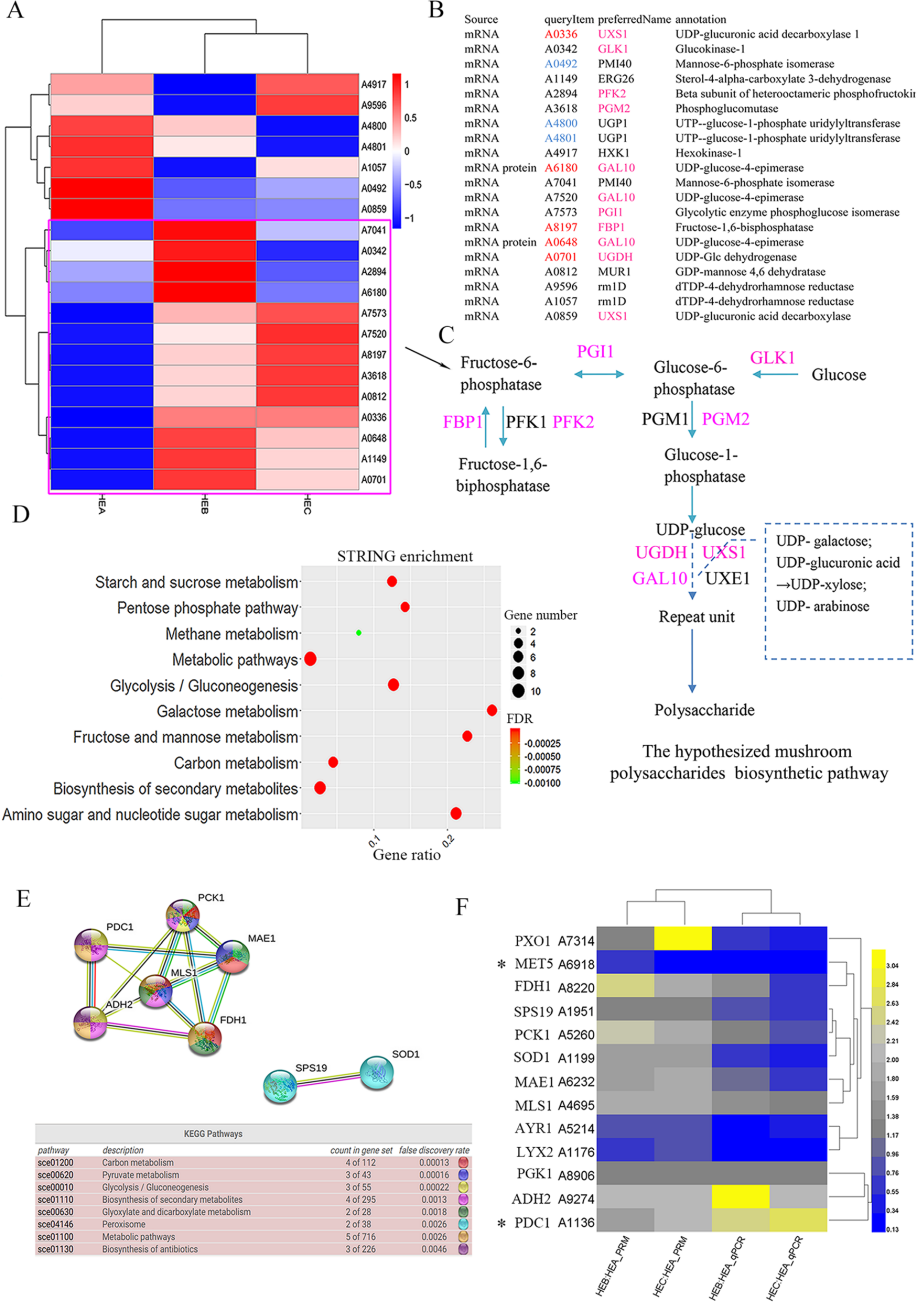
Multi-omics analysis of the MPBP in *H. erinaceus*. **a** Heatmap analysis of expressed mRNA involved in MPBP. The cluster with upregulation in HEB or HEC was marked by purple rectangular. Red represents high expression. Blue represents low expression. **b** The list of DEGs involved in MPBP. Red represents an obvious upregulation; Blue represents an obvious down-regulation. The DEGs were obtained from the transcriptome and proteomics data, respectively. **c** The MPBP modified from reference [14]. Purple represents the upregulated genes in HEB or HEC in the purple rectangular of Fig. 6a. **d** STRING enrichment of the expressed mRNA involved in MPBP. **e** STRING network analysis of the CDC genes. The CDC genes were obtained by the Venn analysis of the co-enriched DEGs using DAVID and STRING enrichment in HEB_vs_HEA and HEC_vs_HEA in Additional file 15. **f** Heatmap clustering of the expression value of the CDC genes using qPCR and PRM. Yellow indicates high expression, and blue indicates low expression. *Represents the genes with the statistical support by qPCR (*P* < 0.05) and PRM (*P* < 0.05)

Twenty homologous genes in *H. erinaceus* were obtained using Blastp (1e-5) of the sequences in yeast based on the hypothesized mushroom polysaccharides biosynthetic pathways (MPBP) according to reference [14]. Heatmap analysis of the mRNA genes involved in MPBP showed a noticeable expressed difference between HEA and the two mutated strains (Fig. 6a), especially the upregulated cluster marked by purple rectangular in HEB or HEC. Among the cluster, FBP1, UGDH, GAL10, and UXS1 had prominent upregulation mRNA expression (Fig. 6b). Only two differentially expressed proteins involved in the MPBP occurred in HEB_vs_HEA (A0648) and HEC_vs_HEA (A6180). The two genes both belonged to GAL10 (UDP-glucose-4-epimerase) involved in the synthesis of polysaccharide repeat units, and they also had the upregulated mRNA and protein expression based on the transcriptome and proteomics data (Fig. 6b). The up-regulation of these genes known to be involved in the biosynthesis of polysaccharides repeat units (Fig. 6c) might explain the higher yield of polysaccharides in the mutated strains.

The twenty homologous genes in MPBP were enriched in amino sugar and nucleotide sugar metabolism, glycolysis/gluconeogenesis, galactose metabolism, fructose, and mannose metabolism (Fig. 6d). These results agreed partially with the 18 co-upregulated proteins in HEB_vs_HEA and HEC_vs_HEA in Fig. 5e, which conformed further to the MPBP activation in mutant strains HEB and HEC.

The DEGs from transcriptome or proteomics were used for the enrichment analysis to obtain the co-enriched and differentially co-expressed (CDC) genes. The CDC genes identified by the Venn analysis of HEB_vs_HEA and HEC_vs_HEA (Additional file 15) were correlated with carbohydrate metabolism, such as carbon metabolism, pyruvate metabolism, and glycolysis/gluconeogenesis (Fig. 6e). This result agreed with the multi-omics analysis of the MPBP in *H. erinaceus* (Fig. 5d). The qPCR and PRM analysis further verified the expressions of the CDC genes. The list of the primers and the peptides followed by PRM was given in Additional file 16 and Additional file 17, respectively. qPCR and PRM analysis confirmed that most of the CDC genes shared a similar expression trend with the transcriptome and proteome data (Fig. 6f), such as the upregulation of MAE1(A6232), MLS1(A4695), PCK1(A5260) in the glyoxylate cycle modules (M00012) and PGK1 (A8906) in the glycolysis module (M00001) (Fig. 5f and Additional file 14). RowCluser analysis showed that MAE1, MLS1, and PCK1 were clustered together based on the PRM and qPCR data (Fig. 6f), representing a similar upregulation trend between mRNA and protein expression. In particular, MET5 (A6918) and PDC1 (A1136) were the two genes with the statistical support by qPCR (*P* < 0.05) and PRM (*P* < 0.05) (Fig. 6f). Interestingly, the M00012 is connected with M00001, which leads to the synthesis of glucose according to the KEGG pathway of carbohydrate metabolism (Additional file 14). The synthesis of glucose is responsible for providing repeat units according to the MPBP modified from reference [14] (Fig. 6c). The CDC genes shared many KEGG pathways involved in carbohydrate metabolism (Fig. 6e), and therefore, their upregulation could increase the activities of carbohydrate metabolism for providing repeat units in polysaccharides production.

### Multi-omics analysis of the glucose signaling regulation dysfunction associated with β-glucan production

**Fig. 7:**
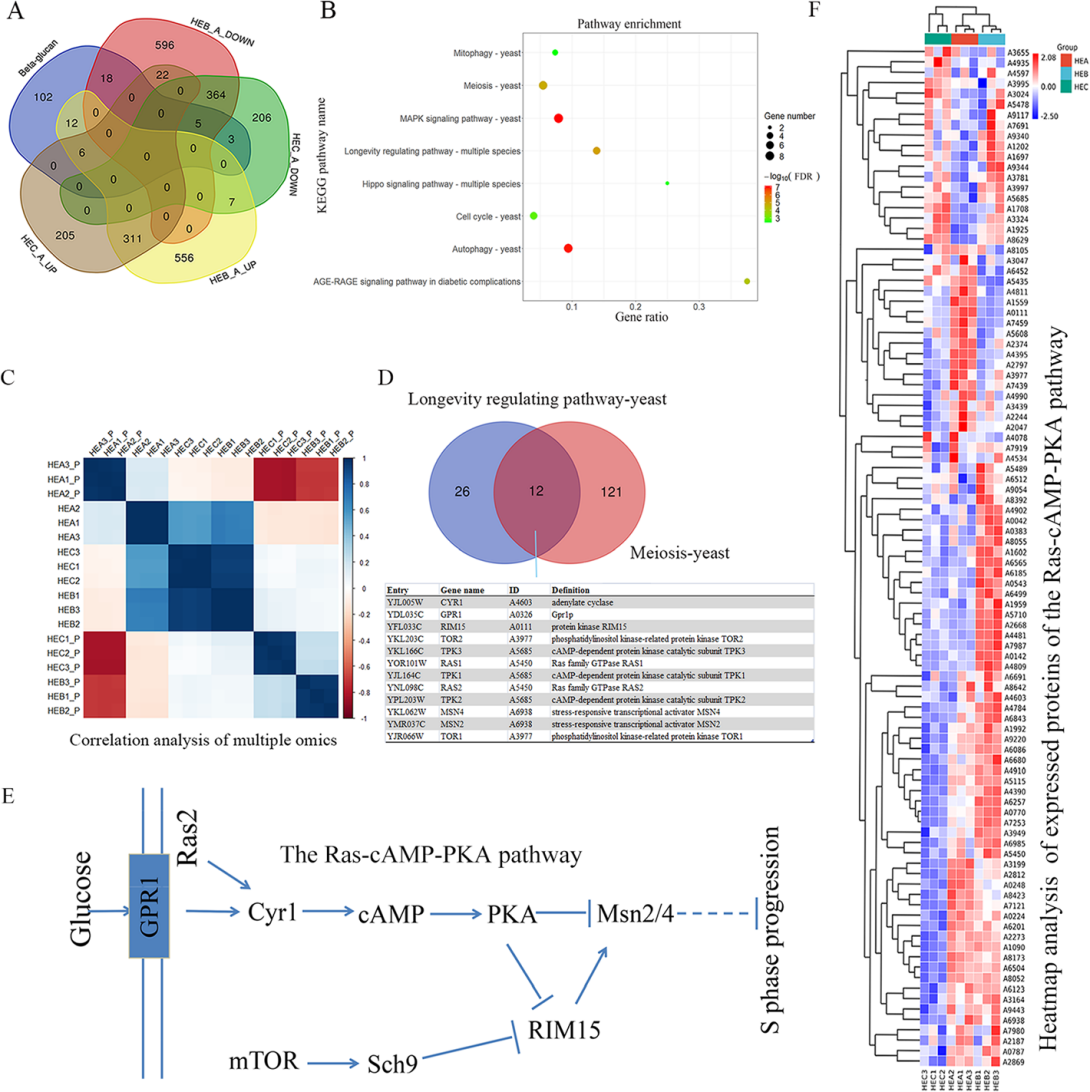
Multi-omics analysis of the regulatory pathway of polysaccharides production in *H. erinaceus*. **a** Venn analysis of the differentially expressed mRNAs involved in the GBP. **b** STRING enrichment of these differentially expressed mRNAs involved in the GBP. **c** Correlation analysis diagram of the transcriptome and proteomics data. One hundred twenty-two differentially expressed mRNAs or proteins that occurred in the comparison groups of HEB_vs_HEA and HEC_vs_HEA (Additional file 6B) were used for the correlation analysis. P represents proteomics data. **d** Venn analysis of the expressed genes in the KEGG pathways of longevity regulating pathway and meiosis-yeast. **e** The RAS-cAMP-PKA pathway in *H. erinaceus*. This pathway was modified from the KEGG pathway of longevity regulation-yeast in Additional file 19. **f** Cluster analysis of expressed protein of the RAS-cAMP-PKA pathway. Red represents high expression. Blue represents low expression

Considering the 92% glucose ratio in the monosaccharide composition and a β-configuration glycosidic bonds in the new polysaccharide fraction produced from HEC (Fig. 1e and f), we further did the multi-omics analysis of the β-glucan biosynthetic process (GBP) to explore the regulatory pathway of glucan production. One hundred forty-six homologous genes in *H. erinaceus* were obtained using Blastp (1e-5) of the sequences in the yeast GBP (GO: 0051274). Heatmap analysis of expressed mRNA genes involved in the GBP showed a noticeable expressed difference between HEA and two mutated strains (Additional file 18A). Venn analysis showed that HEB_vs_HEA had 41 DEGs involved in the GBP bigger than 14 DEGs in HEC_vs_HEA (Fig. 7a). These DEGs were enriched in the MAPK signaling pathway, longevity regulating pathway, cell cycle, meiosis, and AGE-RAGE signaling pathway in diabetic complications, which were closely associated with the signal transduction pathway (Fig. 7b). The pathway of longevity regulation and MAPK signaling pathway were also found in the enrichment analysis using the significantly co-downregulated proteins in HEC_vs_HEA or HEB_vs_HEA (Fig. 5d). Multi-omics analysis suggested that the two pathways played a regulatory role in the GBP of *H. erinaceus*.

Venn analysis of proteomics data showed that only five DEPs involved in the GBP occurred in HEB_vs_HEA or HEC_vs_HEA (Additional file 18B). STRING enrichment of five DEPs showed that they were enriched in signal transduction (GO:0007165). The 13 DEGs involved in the GBP that occurred in HEB_vs_HEA and HEC_vs_HEA were listed in Additional file 18C. Among them, the transcriptome data and proteomics data confirmed the two downregulated genes, A8173 and A5435 (Additional file 18C). STRING annotation showed that A5435 (Sch9) was a serine/threonine-protein kinase involved in ribosome biogenesis, translation initiation, and the regulation of G1 progression. The previous report indicated that Sch9 might converge with the Ras-cAMP pathway downstream of PKA on its effector Rim15 [25]. Like TOR complex 1, Sch9 was required for cytoplasmic retention of Rim15 during exponential growth on glucose-containing medium [26], indicating that Sch9 also mediated the cell growth response to glucose [27].

The correlation analysis of 122 multiple omics data showed that HEB and HEC had a stronger negative correlation with HEA in the proteomic data than in the transcriptome data (Fig. 7c), indicating an apparent difference in protein expression between HEA and the mutated strains. The cluster consisting of steroid biosynthesis, longevity regulation, MAPK signaling pathway, and peroxisome reflected the apparent change in protein expression in HEB_vs_HEA or HEC vs_HEA (Fig. 5d). Multi-omics analysis confirmed that longevity regulation was involved in the GBP (Fig. 7b), shifting our attention to this pathway. Interestingly, the KEGG pathway showed that the genes, such as Ras2 (A5450), Cyr1 (A4603), PKA (A5435), and Msn 2/4 (A6938), in the pathway of longevity regulation-yeast (Additional file 19) shared partially with those in the pathway of meiosis (Additional file 20). Venn analysis showed that 12 genes were present in the longevity regulating pathway and meiosis-yeast (Fig. 7d), and most of them belonged to the genes of the RAS-cAMP-PKA pathway (Fig. 7e).

One hundred fifty homologous genes of 12 sequences in *H. erinaceus* were obtained using Blastp (1e-5). Heatmap analysis confirmed a noticeable decrease in the protein expression profile of the RAS-cAMP-PKA pathway in HEC compared to the HEA and HEB (Fig. 7f). As the RAS-cAMP-PKA pathway acted as a glucose signals pathway [26], the decrease of protein expression in the RAS-cAMP-PKA pathway could lose glucose sensing in *H. erinaceus*. Considering the highly increased rate of polysaccharide content in HEC compared to HEB (Fig. 1b), the downregulated activity of the RAS-cAMP-PKA pathway might be the reason of high β-glucan production.

### A putative model of high polysaccharide production in the mutated *H. erinaceus*

**Fig. 8:**
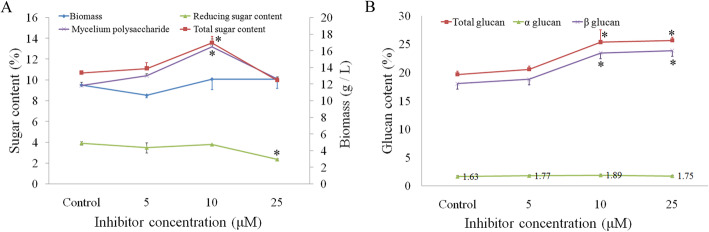
General feature of biomass, sugar, and glucan content in the liquid fermentation of *H. erinaceus* with exogenous CDK inhibitor. **a** Biomass and sugar content in the liquid fermentation of *H. erinaceus* with exogenous inhibitor. **b** Glucan content in the liquid fermentation of *H. erinaceus* with exogenous inhibitor. Data represent the mean ± s.d. (*n* = 3). * indicates *P* < 0.01

The Ras-cAMP-PKA pathway plays a prominent role in responding to glucose availability and initiating the signaling processes that promote cell growth and division [28]. Considering that the Ras-cAMP-PKA pathway eventually connects to S phase progression in the KEGG pathway of meiosis (Additional file 20), the RAS-cAMP-PKA pathway blocking might lead to the inhibition of S phase progression. Exogenous cyclin-dependent kinase (CDK) 1/4/9 P276–00 inhibitor experiment was carried out in wild-type strain HEA, and the results showed that CDK1/4/9 P276–00 increased the intracellular polysaccharide content in *H. erinaceus* (Fig. 8a), especially at 10 μM, which confirmed the regulatory role of RAS-cAMP-PKA pathway in polysaccharide synthesis. Meanwhile, β-glucan content was also increased in a concentration-dependent manner (Fig. 8b), which was consistent with the results of the increased glucose proportion in the monosaccharide composition and production of new β-glucan fraction in the mutant strain HEC (Fig. 1e and f). These observations indicated that the inhibition of S phase progression could lead to the high polysaccharide production in the mutated *H. erinaceus*.

**Fig. 9:**
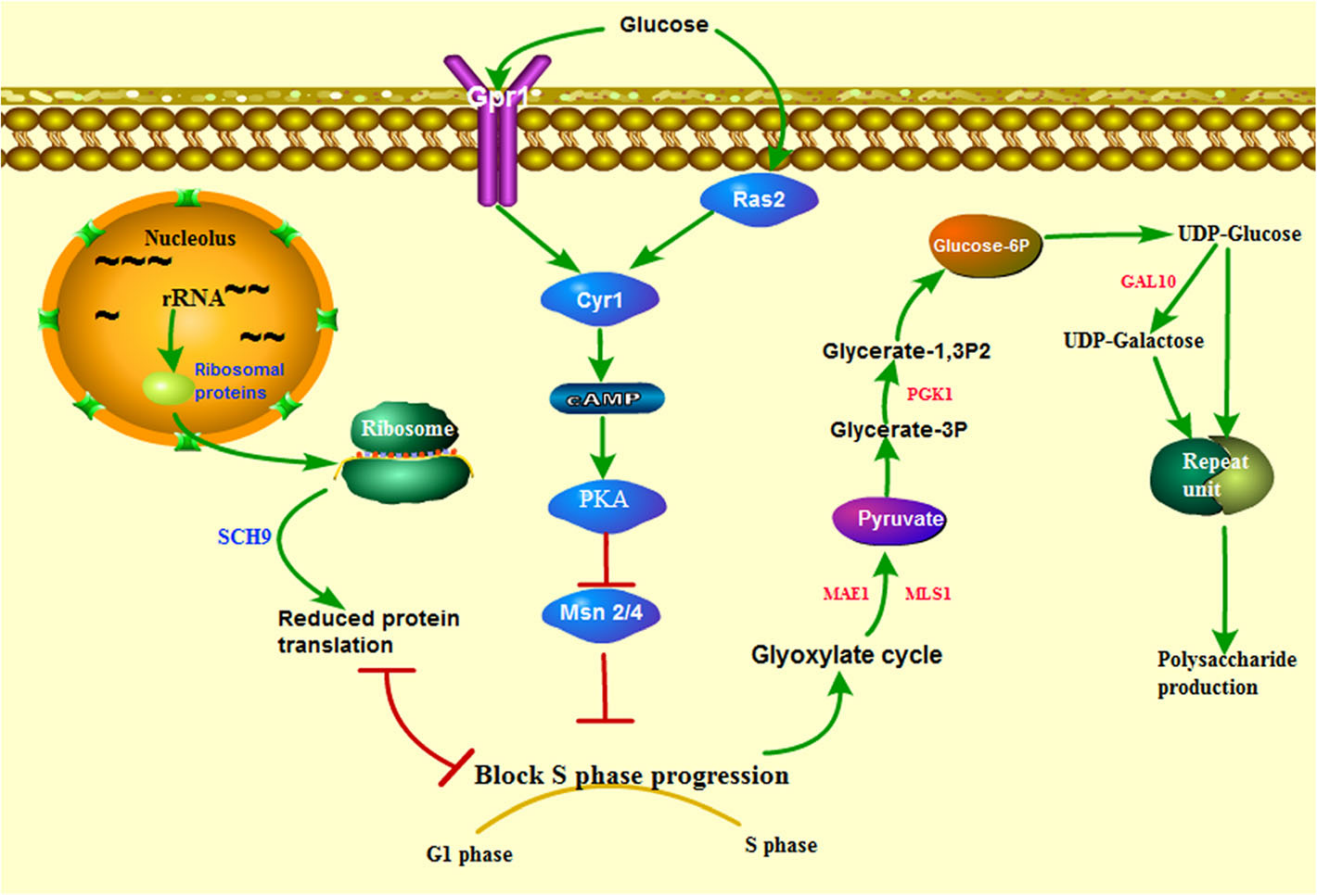
Putative model of high polysaccharide production in the mutated *H. erinaceus*. The Ras-cAMP-PKA pathway could act as glucose signaling regulation. The dysfunction of glucose signaling regulation was due to its reduced expression of the Ras-cAMP-PKA pathway in the mutated *H. erinaceus*, which loses the glucose sensing and cause a long delay in the glucose response, finally resulting in the block of S phase progression. The block of S phase progression might lead to polysaccharide production by increasing the activity of the pathways involved in polysaccharide production. Glyoxylate cycle module and glycolysis module in Fig. 4f participate in the production of the glucose-6p, such as the role of MAF1, MLS1, PGK1 in the two modules. Glucose-6p connects to the pathways involved in producing repeat units through the MPBP in Fig. 6c, such as the role of GAL10 in the conversion from UDP-Glucose to UDP-Galactose. The block of S phase progression might interact with the reduced protein translation in the mutated *H. erinaceus* through SCH9. The image depicted in Fig. 9 is our own

Based on the above data, we proposed a putative model of high polysaccharide production in the mutated *H. erinaceus* (Fig. 9). This model described a disordered process of polysaccharide anabolism. The decreased expression of the RAS-cAMP-PKA pathway might cause a long delay in the glucose response, lose glucose-sensing signaling response, finally inhibiting S phase progression. The block of S phase progression might induce pathways involved in polysaccharide production, such as the elevating activity of the modules of M00012 and M00001 (Fig. 5f and Additional file 14), and the pathways involved in further synthesis of repeating units from glucose-6P (Fig. 6c). The down-regulated ribosomal protein reduced protein translation activity and might interact with the block of S phase progression. Previous researches indicated the role of Sch9 in the Ras-cAMP pathway downstream of PKA through its effector Rim15 [25]. Our observations suggested the role of Sch9 might act in the reduced translation initiation as well as the block of S phase progression (Fig. 9).

The conserved Ras-cAMP-PKA pathway played a central role in the regulation of many biological aspects in eukaryotic organisms [29]. For example, this pathway governs pathogenesis, morphological transitions, nutrient sensing and acquisition, sexual reproduction, and stress responses in fungi [29–32]. In *S. cerevisiae*, the transcriptional responses to glucose are triggered by various PKA mediated pathways, alone or in combination [33]. Multi-omics analysis of our study indicated the disfunction of the RAS-cAMP-PKA pathway in the ARTP mutated strain plays an important role in the stimulating of polysaccharides, especially β-glucan production.

## Conclusion

In this study, we reported the original strain genome of *H. erinaceus* using a single molecule, real-time sequencing technology, and multi-omics analysis was carried out between mutant strains obtained by ARTP mutagenesis and the original strain. Multi-omics analysis indicated that the increased carbohydrate metabolism and the production of glucose-6P constituted the basis of high polysaccharide yield in ARTP mutated strain. Furthermore, the RAS-cAMP-PKA pathway’s decreased activity might promote high polysaccharide and β-glucan production through the block of S phase progression. The study revealed the mechanism of the increased polysaccharide synthesis induced by ARTP mutagenesis associated with carbohydrate metabolism and glucose signaling regulation dysfunction and provided the critical theoretical and practical basis for polysaccharide production in *H. erinaceus*.

## Methods

### Strains and culture conditions

*H. erinaceus* strain 0605 which named HEA was obtained from the Herbarium of Edible Fungi Culture Collection Center Branch of the China Culture Collection of Agricultural Microorganisms (Shanghai, China). It was incubated on PDA (Potato Dextrose Agar, BD, USA) slants at 26 °C.

ARTP (Atmospheric Room Temperature Plasma) was employed to generate *H. erinaceus* mutants 414 (HEB) and 321 (HEC) with high polysaccharide yield, in our previous research [34]. Protoplasts preparation of *H. erinaceus* fermentation mycelium was the first step, more detailed breeding methods and operation steps of strain selection, refer to our published articles [6, 34].

### Analysis of polysaccharide content and structural characteristics of different polysaccharide fractions from original and mutated strains

The total polysaccharide content of fermented mycelia from the original strain and mutated strains was determined using the phenol-sulfuric acid method, according to Dubois, M et al. [35]. Molecular weight distribution patterns among different polysaccharide fractions between original strain and mutated strain were determined by high-performance size-exclusion chromatography (HPSEC) equipped with a refractive index detector (RI) and a UV detector (Waters, Milford, Ma, USA) for assessing concentration, a multiple angle laser light scattering detector (MALLS, Wyatt Technology, Santa Barbara, CA, USA) for direct molecular determination. Chromatographic analysis column selected TSK PWXL6000 (7.8 × 300 nm) (Tosoh, Toyosawa, Fukuroi, Shizuoka, Japan) gel filtration column linked a TSK PWXL4000 (7.8 × 300 nm) gel filtration, which was eluted with phosphate buffer at a flow rate of 0.5 mL/min. The monosaccharide compositions were determined by a high-performance anion-exchange chromatography (HPAEC) system (Dionex ICS-2500, Dionex, Sunnyvale, CA, USA) equipped with a CarboPac™ PA20 column (3 mm × 150 mm, Dionex, Sunnyvale, CA, USA) and a pulsed amperometric detector (Dionex, Sunnyvale, CA, USA). The column was eluted with 2 mM NaOH (0.45 mL/min) followed by 0.05 to 0.2 M NaAc at 30 °C. The monosaccharide compositions were determined using d-Gal, d-Glc, d-Ara, l-Fuc, l-Rha, d-Man, d-Xyl, d-Fru, d-Rib, d-GluA, and d-GalA (Sigma-Aldrich St. Louis, MO, USA) as the standards. Infrared spectra of the different polysaccharide fractions were recorded with an FT-IR spectrometer (Thermo Fisher Scientific, Waltham, MA, USA) in the range 4000 ~ 400 cm^− 1^ using the KBr disk method [36].

### Genome sequencing and assembly

The genomic DNA of *H. erinaceus* HEA was analyzed from the liquid fermentation mycelia. The whole-genome sequencing was ready for this species (accession number: JABWEG000000000). Firstly, the monokaryon of HEA strain was obtained by protoplast preparation and regeneration. The monokaryon strain was incubated on PDA slants at 26 °C. The mycelia seed culture was carried out in a 250-mL flask containing 100 mL potato dextrose broth (PDB, BD, USA) medium at 26 °C on a rotary shaker incubator (150 rev min^− 1^) for 7 d. The fermentation culture was performed in a 500-mL flask containing 200 mL medium by inoculating 10% (v/v) seed culture medium homogenized by homogenizer, and carried out on a rotary shaker incubator (150 rev/min) at 26 °C for 6 d. Fermentation medium consisted of 24 g/L PDB, 1 g/L KH_2_PO_4_, 1 g/L MgSO_4_·7H_2_O, respectively. The mycelia were obtained by centrifuging at 12,000 g, 4 °C for 10 min, washing the mycelia twice with distilled water, and then transferred to EP tube and frozen by dry ice, − 70 °C storage for DNA extraction.

A total of 0.1 g mycelium was first lysed with GP1 lysate. The impurities in the lysate are removed by phenol/chloroform/isoamyl alcohol extraction, and finally, the genomic DNA was obtained after isopropanol precipitation. Details about DNA extraction are available in “genome DNA extraction and assembly” in additional material. The harvested DNA was detected by the agarose gel electrophoresis and quantified by Qubit® 2.0 Fluorometer (Thermo Scientific). The genome of *H. erinaceus* was sequenced by PacBio single-molecule, real-time (SMRT) sequencing technology. SMRT sequencing technology as the 3rd generation, overcomes the GC bias, long in read length, by taking advantage of the long-read and single molecular sequencing capability. Sequencing was performed at the Beijing Novogene Bioinformatics Technology Co., Ltd. The low-quality reads were filtered by the SMRT Link v5.0.1, and the filtered reads were assembled to generate one contig without gaps [37]. Details about genome assembly could be found in “genome DNA extraction and assembly” in additional material.

Augustus 2.7 program was used to retrieve the related coding gene [38]. The interspersed repetitive sequences were predicted using the RepeatMasker (http://www.repeatmasker.org/). The tandem Repeats were analyzed by the TRF (Tandem repeats finder). Transfer RNA (tRNA) genes were predicted by the tRNAscan-SE. Ribosome RNA (rRNA) genes were analyzed by the rRNAmmer. sRNA, snRNA and miRNA were predicted by BLAST against the Rfam database.

### Gene prediction and annotation

We used seven databases to predict gene functions. They were respective GO (Gene Ontology), KEGG (Kyoto Encyclopedia of Genes and Genomes), KOG (Clusters of Orthologous Groups), NR (Non-Redundant Protein Database databases), TCDB (Transporter Classification Database), P450, and, Swiss-Prot. A whole-genome Blast search (E-value less than 1e^− 5^, minimal alignment length percentage larger than 40%) was performed against the above seven databases. The secretory proteins were predicted by the Signal P database. Meanwhile, the secondary metabolism gene clusters were analyzed by the antiSMASH [39]. We used the PHI (Pathogen Host Interactions), DFVF (database of fungal virulence factors) to perform the pathogenicity and drug resistance analyses. Carbohydrate-Active enzymes were predicted by the Carbohydrate-Active enzymes Database. BLAST comparison of predictive genes with each functional database (BLASTp, evalue ≤1e-5) was performed for sequence annotation.

### Phylogenomic tree construction and family size analysis

Together with *H. erinaceus*, 29 fungal species assigned mainly to the Basidiomycota and Ascomycota were used in the phylogenomic analysis. Genomic data sources for these species could be found in references [40]. Single-copy orthologous protein sequences from the genomes of 29 species were obtained using our custom Perl program. The tandem concatenated sequences consisting of the single-copy orthologous sequences from the 29 species were then used to construct a phylogenomic tree maximum-likelihood. Sequences were aligned using Clustalw2 at default parameters. Maximum likelihood trees were inferred using PhyML v3.0 [41] with the LG model [42]. Clade support was calculated using SH-like approximate likelihood ratio tests (aLRT) [43]. PhyML analyses were performed using NNI tree topology searches with estimated Gamma shape parameters.

Total protein sequences from 12 representative species genomic databases were used to study the change of the family size. Multigene families were generated from all the predicted proteins of selected genomes using SCPS tools [44] with default settings (Blastp, cut-off e-value <1e^− 7^). The obtained multigene families were then analyzed for evolutionary changes in protein family size (> 1) using the CAFE program [45].

### RNA preparation, extraction, sequencing, and transcriptome analysis

The total RNA was extracted from the liquid fermentation mycelia of *H. erinaceus* (HEA, HEB, and HEC), which was consistent with the genomic DNA extraction material, using RNAprep Pure Plant Kit (Polysaccharides&Polyphenolics-rich) according to the manufacturer’s specifications (TIANGEN, Beijing, China). The culture method of liquid fermentation mycelia of *H. erinaceus* was consistent with the previous genomic DNA extraction material. There were three biological replicates in this study. A total amount of 1 μg RNA per sample was used as input material for the RNA sample preparations. Sequencing libraries were generated using NEBNext® Ultra™ RNA Library Prep Kit for Illumina® (NEB, USA) following the manufacturer’s recommendations, and index codes were added to attribute sequences to each sample. PCR products were purified (AMPure XP system), and library quality was assessed on the Agilent Bioanalyzer 2100 system. The clustering of the index-coded samples was performed on a cBot Cluster Generation System using TruSeq PE Cluster Kit v3-cBot-HS (Illumia) according to the manufacturer’s instructions. After cluster generation, the library preparations were sequenced on an Illumina Hiseq platform, and 125 bp/150 bp paired-end reads were generated.

Raw data (raw reads) of fastq format were processed through in-house Perl scripts for obtaining the clean data with high quality. Hisat2 v2.0.4 was used for reads mapping to the *H. erinaceus* reference genome with default parameters [46]. Differential expression analysis was performed using the DESeq2 R package (1.16.1) with default parameters [47]. The resulting *P*-values were adjusted using the Benjamini and Hochberg’s approach for controlling the false discovery rate. Genes with an adjusted *P*-value < 0.05 found by DESeq were assigned as differentially expressed. The details are available in “data analysis for transcriptome analysis” in additional material.

Gene Ontology (GO) enrichment analysis of differentially expressed genes with a corrected *P*-value less than 0.05 was implemented by the GOseq R package [48]. We used KOBAS software to test the statistical enrichment of differential expressed genes in KEGG pathways [49]. Protein-protein interaction analysis and functional enrichment analysis of differentially expressed genes were based on the STRING database using yeast as the reference species [50].

### Protein extraction for proteomic analysis

One hundred micrograms protein extraction of *H. erinaceus* mycelia from liquid fermentation was used for protein enzymatic hydrolysis, according to the FASP method [51]. There were three biological replicates in this study. The concentration of this sample was determined by the method of BCA protein concentration [52]. The solutions were collected and lyophilized for TMT labeling. The labeling peptides solutions were lyophilized and stored at − 80 °C. RP separation was performed on an 1100 HPLC System (Agilent) using an Agilent Zorbax Extend RP column. The separated peptides were lyophilized for MS detection. All analyses were performed by a Q-Exactive HF mass spectrometer (Thermo, USA) equipped with a Nanospray Flex source (Thermo, USA). Proteome Discoverer (v.2.2) was used to search all of the Q Exactive raw data thoroughly against the sample protein database. Database searches were performed with Trypsin digestion specificity. Alkylation on cysteine was considered as fixed modifications in the database searching. For the protein quantification method, TMT10-plex was selected. Only proteins identified at global FDR ≤ 1% and unique peptides ≥1 were considered for protein lists and further downstream analysis. For a protein to be determined as differentially expressed, the *p*-values of the protein quantitation should be less than 0.05 and fold change ≥1.2. The OmicsBean cloud platform was used for the bioinformatics analysis of differentially expressed proteins. R language is used for the heat map clustering of the expression pattern. The details about the proteomic analysis could be found in additional material.

### Detection of quantitative real-time PCR

Total RNA was extracted from *H. erinaceus* mycelia from liquid fermentation using RNAprep Pure Plant Kit (Polysaccharides&Polyphenolics-rich) according to the manufacturer’s specifications. The yield of RNA was determined using a NanoDrop 2000 spectrophotometer (Thermo Scientific, USA), and the integrity was evaluated using agarose gel electrophoresis stained with ethidium bromide.

Quantification was performed with a two-step reaction process: reverse transcription (RT) and PCR. Each RT reaction has two steps. The first step is 0.5 μg RNA, 2 μL of 4 × gDNA wiper Mix, add Nuclease-free H_2_O to 8 μL. Reactions were performed in a GeneAmp® PCR System 9700 (Applied Biosystems, USA) for 2 min at 42 °C. The second step is to add 2 μL of 5 × HiScript II Q RT SuperMix IIa. Reactions were performed in a GeneAmp® PCR System 9700 (Applied Biosystems, USA) for 10 min at 25 °C; 30 min at 50 °C; 5 min at 85 °C. The 10 μL RT reaction mix was then diluted × 10 in nuclease-free water and held at − 20 °C. Real-time PCR was performed using LightCycler® 480 II Real-time PCR Instrument (Roche, Swiss) with 10 μL PCR reaction mixture that included 1 μL of cDNA, 5 μL of 2× QuantiFast® SYBR® Green PCR Master Mix (Qiagen, Germany), 0.2 μL of forward primer, 0.2 μL of reverse primer and 3.6 μL of nuclease-free water. Reactions were incubated in a 384-well optical plate (Roche, Swiss) at 95 °C for 5 min, followed by 40 cycles of 95 °C for 10 s, 60 °C for 30 s. Each sample was run in triplicate for analysis. At the end of the PCR cycles, the melting curve analysis was performed to validate the specific generation of the expected PCR product. The primer sequences listed in Additional file 16 were designed in the laboratory and synthesized by Generay Biotech (Generay, PRC). The expression levels of mRNAs were normalized to 18S rRNA and were calculated using the 2^-ΔΔCt^ method [53].

### PRM targeted quantitative proteomic analysis

Refer to protein extraction in proteomics extraction, 50 μL 100 mM TEAB was added and centrifuged. The pH of all solutions was adjusted to 1–3 by H_3_PO_4_ for desalination. The digested peptides were desalted by C18-Reverse-Phase SPE Column. The iRT standard peptides were dissolved by relative buffer according to the user manual [2xiRT Kit Quick Reference Card]. Briefly, the iRT standard was dissolved to 10× and stored at 4 °C. Before LC-MS injection, the 10× iRT standard peptide mix was added to injection-ready peptide samples (iRT:sample = 1:10 v/v). The sample mix was first run in DDA mode and analyzed with MaxQuant [54] to obtain the retention times of the peptides which were used to set up a scheduled PRM assay. Briefly, the DDA raw files were analyzed with MaxQuant (version 1.3.0.5) software using default settings. The minimal peptide length was set to 7. Trypsin was used as a digestion enzyme. Search criteria included carbamidomethylation of cysteine as a fixed modification, oxidation of methionine, and acetyl (protein N terminus) as variable modifications. Up to two missed cleavages were allowed. The mass tolerance for the precursor was 20 ppm and 0.5 Da for MS/MS respectively, and for the fragment ions was 50 ppm. The DDA files were searched against the human UniProt fasta database (July 2018) in which the Biognosys iRT peptide sequences (11 entries) were added. The identifications were filtered to obtain FDR of 1% at the peptide and the protein level. A list of peptides from DDA analysis was prepared for Parallel Reaction Monitoring (PRM) validation (at least 2 peptides per protein). Samples were loaded on a RSLC, 75 μm × 15 cm, nanoViper, C18, 3 μm, 100Ȧ column (Acclaim, PepMap) retrofitted to an EASY-Spray source with a flow rate of 300 nl/min (buffer A: HPLC H_2_O, 0.1% formic acid, buffer B: 80% ACN, 0.1% formic acid). A 90 min gradient was performed as follows: 0 ~ 55 min, 8–30% B; 55 ~ 79 min, 30–50% B; 79 ~ 80 min, 50–100% B; 80 ~ 90 min, 100% B. Peptides were transferred to the gaseous phase with positive ion electrospray ionization at 2.1 kV. For DDA, the top 10 precursors were acquired between 300 and 1600 m/z with a 3 m/z isolation window, dynamic exclusion of 30 s, normalized collision energy (NCE) of 25, and resolution of 70,000. For PRM, precursors were targeted in a 1.2 m/z isolation window around the m/z of interest. Precursors were fragmented in HCD mode with NCE energy of 25. MS1 was performed at a 70,000 resolution, an AGC target of 1e6 and a maximum injection time was 50 ms; MS/MS was performed at 17,500 resolution, an AGC target of 2e5 and a maximum injection time was 50 ms. Spectra were analyzed using Skyline [55] with manual validation. Skyline quantitation data was exported to excel, and the quantitation data was normalized against the TIC of the MS runs. The list of the peptides followed by PRM is given in Additional file 17.

### Exogenous CDK inhibitor experiment

Riviciclib hydrochloride (P276–00, Selleck) is a novel CDK inhibitor, which inhibits CDK1/4/9. Referring to previous references [56], CDK1/4/9 P276–00 was used to block S phase progression during the fermentation process of the original strain HEA in this study. The culture method of liquid fermentation mycelia was based on our published article [22]. P276–00 was dissolved in sterile ddH_2_O and added to the fermentation broth on the third day during mycelia fermentation at final concentrations of 5 μM, 10 μM, 25 μM, and 50 μM, respectively. In the blank control, the inhibitor was replaced by an equal amount of water. Three parallel experiments were carried out simultaneously. *H. erinaceus* mycelia were freeze-dried after 7 days cultivation to get a constant dry biomass weight. The total polysaccharide production was assayed using the phenol-sulfuric acid method, according to the published literature [35]. Reducing sugar content was determined using the DNS method according to reference [57]. The glucan content in the sample was determined according to the yeast beta-glucan kit provided by megazyme international Ireland limited.

### Statistical analysis

Statistical analysis was carried out using SPSS 26.0 software (SPSS Inc., Chicago, United States). One-way analysis of variance (ANOVA) was adopted to compare the significant differences among all groups using Tukey’s test. A probability level of *p* < 0.05 was set as statistical significance. Data were presented as mean ± standard deviation (SD) of at least three independent experiments.

## Supplementary Information

**Additional file 1.** KEGG pathway enrichment of the significantly upregulated genes in HEB_vs_HEA.

**Additional file 2.** KEGG pathway enrichment of the significantly upregulated genes in HEC_vs_HEA.

**Additional file 3.** KEGG pathway enrichment of the significantly downregulated genes in HEB_vs_HEA.

**Additional file 4.** KEGG pathway enrichment of the significantly downregulated genes in HEC_vs_HEA.

**Additional file 5. **Transcriptome analysis of *H. erinaceus*. (A) Venn diagram of significantly upregulated genes from the comparison group of HEC_vs_HEA and HEB_vs_HEA. (B) STRING enrichment analysis of significantly co-upregulated genes. (C) Venn diagram of significantly downregulated genes from the comparison group of HEC_vs_HEA and HEB_vs_HEA. (D) STRING enrichment analysis of significantly co-downregulated genes.

**Additional file 6. **Proteomics analysis of *H. erinaceus*. (A) PCA of the expressed proteins. (B) Venn diagram analysis of significantly expressed proteins from the comparison group of HEC_vs_HEA and HEB_vs_HEA**.** (C) Heatmap clustering of the significantly differentially expressed proteins.

**Additional file 7.** The expressed proteins of the comparison group of HEC_vs_HEA and HEB_vs_HEA.

**Additional file 8.** The significantly expressed proteins of the comparison group of HEB_vs_HEA.

**Additional file 9.** The significantly expressed proteins of the comparison group of HEC_ vs_HEA.

**Additional file 10.** KEGG pathway enrichment of the significantly upregulated proteins in HEB_vs_HEA.

**Additional file 11.** KEGG pathway enrichment of the significantly downregulated proteins in HEB_vs_HEA.

**Additional file 12.** KEGG pathway enrichment of the significantly upregulated proteins in HEC_vs_HEA.

**Additional file 13.** KEGG pathway enrichment of the significantly downregulated proteins in HEC_vs_HEA.

**Additional file 14.** The KEGG mapping of the enriched pathway modules of carbohydrate metabolism in HEB_vs_HEA. Pink shading lines marked the glyoxylate cycle modules (M00012) and the glycolysis module (M00001). Red lines represent the significantly upregulated proteins. Blue lines represent the significantly downregulated proteins. These images are obtained by KEGG [23]. We have obtained the appropriate copyright permission to modify the KEGG pathways depicted in Additional file 14.

**Additional file 15.** Venn analysis of the CDC genes in the comparison groups of HEB_vs_HEA and HEC_vs_HEA. (A) Venn analysis of CDC genes based on the DAVID enrichment of the significantly upregulated mRNAs and proteins. (B) Venn analysis of the CDC genes based on the STRING enrichment of the significantly upregulated mRNAs and proteins. (C) Venn analysis of the CDC genes based on the DAVID enrichment of the significantly downregulated mRNAs and proteins. (D) Venn analysis of the CDC genes based on the DAVID enrichment of the significantly downregulated mRNAs and proteins. Note: HM0 = HEA, HM1 = HEC, HM2 = HEB.

**Additional file 16.** The primer sequences for real-time quantitative RT-PCR. 18 s rRNA was used as reference genes.

**Additional file 17.** Retention time and quantitative value of iRT peptide and target peptide in PRM.

**Additional file 18. **Multi-omics analysis of the GBP in *H. erinaceus*. (A) Heatmap analysis of expressed mRNA genes involved in the GBP. Red represents high expression. Blue represents low expression. (B) Venn analysis of the differentially expressed proteins involved in the GBP. (C) The list of DEGs occurred in HEB_vs_HEA and HEC_vs_HEA involved in the GBP. Red represents an apparent upregulation; Blue represents an apparent downregulation. The DEGs were obtained from transcriptome and proteomics, respectively.

**Additional file 19.** The KEGG mapping of the enriched pathway of longevity regulation in HEC_vs_HEA. The red box represents the significantly upregulated proteins. The blue box represents the significantly downregulated proteins. These images are obtained by KEGG [23]. We have obtained the appropriate copyright permission to modify the KEGG pathways depicted in Additional file 19.

**Additional file 20.** The KEGG mapping of the enriched pathway of meiosis in HEC_vs_HEA. The red line represents the significantly upregulated proteins. The blue line represents the significantly downregulated proteins. The red box represents the significantly upregulated proteins. The blue box represents the significantly downregulated proteins. These images are obtained by KEGG [23]. We have obtained the appropriate copyright permission to modify the KEGG pathways depicted in Additional file 20.

## Data Availability

Genome sequencing of *H. erinaceus* 0605 generated for this study have been submitted to the NCBI (https://www.ncbi.nlm.nih.gov) under the accession number of JABWEG000000000. RNA-seq data generated for this study have been submitted to the NCBI under the accession number of GSE165264 (https://www.ncbi.nlm.nih.gov/geo/query/acc.cgi?acc=GSE165264). All other data are available from the corresponding author upon reasonable request.

## Abbreviations

ARTP: Atmospheric pressure room temperature plasma
UGP: UDP glucose pyrophosphorylase
GLS: β-1,3-glucan synthase
PGM: α-phosphoglucomutase
PMI: Phosphomannose isomerase
PGI: Phosphoglucose isomerase
DEGs: Differentially expressed genes
DEPs: Differentially expressed proteins
MPBP: The hypothesized mushroom polysaccharides biosynthetic pathways
CDC: Co-enriched and differentially co-expressed
GBP: β-glucan biosynthetic process
CDK: Cyclin-dependent kinase

## References

[1] Sumerford DV, Head GP, Shelton A, Greenplate J, Moar W (2013). Field-evolved resistance: assessing the problem and ways to move forward. J Econ Entomol.

[2] Li QZ, Wu D, Chen X, Zhou S, Liu Y, Yang Y, Cui F (2015). Chemical compositions and macrophage activation of polysaccharides from Leon's mane culinary-medicinal mushroom Hericium erinaceus (higher Basidiomycetes) in different maturation stages. Int J Med Mushrooms.

[3] Wu D, Tang C, Liu Y, Li Q, Wang W, Zhou S, Zhang Z, Cui F, Yang Y (2019). Structural elucidation and immunomodulatory activity of a β-D-glucan prepared by freeze-thawing from Hericium erinaceus. Carbohydr Polym.

[4] Zan X, Cui F, Li Y, Yang Y, Wu D, Sun W, Ping L (2015). Hericium erinaceus polysaccharide-protein HEG-5 inhibits SGC-7901 cell growth via cell cycle arrest and apoptosis. Int J Biol Macromol.

[5] Wang XY, Zhang DD, Yin JY, Nie SP, Xie MY (2019). Recent developments in Hericium erinaceus polysaccharides: extraction, purification, structural characteristics and biological activities. Crit Rev Food Sci Nutr.

[6] Zhu L, Wu D, Zhang H, Li Q, Zhang Z, Liu Y, Zhou S, Wang W, Li Z, Yang Y. Effects of Atmospheric and Room Temperature Plasma (ARTP) mutagenesis on physicochemical characteristics and immune activity in vitro of hericium erinaceus polysaccharides. Molecules. 2019;24(2):262. 10.3390/molecules24020262. 10.3390/molecules24020262 PMC6358873 30641994

[7] Cor D, Knez Z, Knez Hrncic M. Antitumour, antimicrobial, antioxidant and antiacetylcholinesterase effect of ganoderma lucidum terpenoids and polysaccharides: a review. Molecules. 2018;23(3):649. 10.3390/molecules23030649. 10.3390/molecules23030649 PMC6017764 29534044

[8] He X, Wang X, Fang J, Chang Y, Ning N, Guo H, Huang L, Huang X, Zhao Z (2017). Structures, biological activities, and industrial applications of the polysaccharides from Hericium erinaceus (Lion's mane) mushroom: a review. Int J Biol Macromol.

[9] Zhang J, Wen C, Duan Y, Zhang H, Ma H (2019). Advance in Cordyceps militaris (Linn) link polysaccharides: isolation, structure, and bioactivities: a review. Int J Biol Macromol.

[10] He X, Wang X, Fang J, Chang Y, Ning N, Guo H, Huang L, Huang X, Zhao Z (2017). Polysaccharides in Grifola frondosa mushroom and their health promoting properties: a review. Int J Biol Macromol.

[11] Li HJ, Zhang DH, Yue TH, Jiang LX, Yu X, Zhao P, Li T, Xu JW (2016). Improved polysaccharide production in a submerged culture of Ganoderma lucidum by the heterologous expression of Vitreoscilla hemoglobin gene. J Biotechnol.

[12] Peng L, Qiao S, Xu Z, Guan F, Ding Z, Gu Z, Zhang L, Shi G (2015). Effects of culture conditions on monosaccharide composition of Ganoderma lucidum exopolysaccharide and on activities of related enzymes. Carbohydr Polym.

[13] Zhu ZY, Liu XC, Dong FY, Guo MZ, Wang XT, Wang Z, Zhang YM (2016). Influence of fermentation conditions on polysaccharide production and the activities of enzymes involved in the polysaccharide synthesis of Cordyceps militaris. Appl Microbiol Biotechnol.

[14] Wang Q, Wang F, Xu Z, Ding Z. Bioactive mushroom polysaccharides: a review on monosaccharide composition, biosynthesis and regulation. Molecules. 2017;22(6):955. 10.3390/molecules22060955. 10.3390/molecules22060955 PMC6152739 28608797

[15] Tan X, Sun J, Ning H, Qin Z, Miao Y, Sun T, Zhang X (2018). De novo transcriptome sequencing and comprehensive analysis of the heat stress response genes in the basidiomycetes fungus Ganoderma lucidum. Gene.

[16] Zhang N, Tang Z, Zhang J, Li X, Yang Z, Yang C, Zhang Z, Huang Z (2019). Comparative transcriptome analysis reveals the genetic basis underlying the biosynthesis of polysaccharides in Hericium erinaceus. Bot Stud.

[17] Chen J, Zeng X, Yang YL, Xing YM, Zhang Q, Li JM, Ma K, Liu HW, Guo SX (2017). Genomic and transcriptomic analyses reveal differential regulation of diverse terpenoid and polyketides secondary metabolites in Hericium erinaceus. Sci Rep.

[18] Huang Y, Wu X, Jian D, Zhan Y, Fan G (2015). De novo transcriptome analysis of a medicinal fungi Phellinus linteus and identification of SSR markers. Biotechnol Biotechnol Equip.

[19] Shu S, Chen B, Zhou M, Zhao X, Xia H, Wang M (2013). De novo sequencing and transcriptome analysis of Wolfiporia Cocos to reveal genes related to biosynthesis of triterpenoids. PLoS One.

[20] Yang F, Xu B, Zhao S, Li J, Yang Y, Tang X, Wang F, Peng M, Huang Z (2012). De novo sequencing and analysis of the termite mushroom (Termitomyces albuminosus) transcriptome to discover putative genes involved in bioactive component biosynthesis. J Biosci Bioeng.

[21] Wang G, Xu L, Yu H, Gao J, Guo L (2019). Systematic analysis of the lysine succinylome in the model medicinal mushroom Ganoderma lucidum. BMC Genomics.

[22] Tian-Tian S, Di W, He-Nan Z, Wen-Han W, Yan-Fang L, Qiao-Zhen L, Yan Y (2018). Physicochemical properties and immunological activities in vitro of mycelia polysaccharides from Hericium erinaceus mutants induced by atmospheric and room temperature plasma. Mycosystema.

[23] Kanehisa M, Goto S (2000). KEGG: Kyoto encyclopedia of genes and genomes. Nucleic Acids Res.

[24] Cheng X, Zheng X, Zhou X, Zeng J, Ren Z, Xu X, Cheng L, Li M, Li J, Li Y (2016). Regulation of oxidative response and extracellular polysaccharide synthesis by a diadenylate cyclase in Streptococcus mutans. Environ Microbiol.

[25] Roosen J, Engelen K, Marchal K, Mathys J, Griffioen G, Cameroni E, Thevelein JM, De Virgilio C, De Moor B, Winderickx J (2005). PKA and Sch9 control a molecular switch important for the proper adaptation to nutrient availability. Mol Microbiol.

[26] Swinnen E, Wanke V, Roosen J, Smets B, Dubouloz F, Pedruzzi I, Cameroni E, De Virgilio C, Winderickx J (2006). Rim15 and the crossroads of nutrient signalling pathways in Saccharomyces cerevisiae. Cell Div.

[27] Broach JR (2012). Nutritional control of growth and development in yeast. Genetics.

[28] Busti S, Coccetti P, Alberghina L, Vanoni M (2010). Glucose signaling-mediated coordination of cell growth and cell cycle in Saccharomyces cerevisiae. Sensors (Basel).

[29] D'Souza CA, Heitman J (2001). Conserved cAMP signaling cascades regulate fungal development and virulence. FEMS Microbiol Rev.

[30] Hogan DA, Sundstrom P (2009). The Ras/cAMP/PKA signaling pathway and virulence in Candida albicans. Future Microbiol.

[31] Kronstad JW, Hu G, Choi J (2011). The cAMP/protein kinase a pathway and virulence in Cryptococcus neoformans. Mycobiology.

[32] Caza M, Kronstad JW (2019). The cAMP/protein kinase a pathway regulates virulence and adaptation to host conditions in Cryptococcus neoformans. Front Cell Infect Microbiol.

[33] Livas D, Almering MJ, Daran JM, Pronk JT, Gancedo JM (2011). Transcriptional responses to glucose in Saccharomyces cerevisiae strains lacking a functional protein kinase a. BMC Genomics.

[34] Yang S, Yang Y, Li Q, Di W, Yang R, Wang W, Zhang H (2019). Mutation breeding of screening high-yielding polysaccharide Hericium erinareus by atmospheric and room temperature plasma. Acta Agric Shanghai.

[35] Dubois M, Gilles HA, Hamilton JK, Rebers PA, Smith F (1956). Colorimetric method for determination of sugars and related substances. Anal Chem.

[36] Wu Y, Cui SW, Tang J, Wang Q, Gu X (2007). Preparation, partial characterization and bioactivity of water-soluble polysaccharides from boat-fruited sterculia seeds. Carbohydr Polym.

[37] Li R, Zhu H, Ruan J, Qian W, Fang X, Shi Z, Li Y, Li S, Shan G, Kristiansen K (2010). De novo assembly of human genomes with massively parallel short read sequencing. Genome Res.

[38] Stanke M, Diekhans M, Baertsch R, Haussler D. Using native and syntenically mapped cDNA alignments to improve de novo gene finding. Bioinformatics (Oxford, England). 2008;24(5):637-44. 10.1093/bioinformatics/btn013 18218656

[39] Medema MH, Blin K, Cimermancic P, Jager VCL, Zakrzewski P, Fischbach MA, Weber T, Takano E, Breitling R (2011). antiSMASH: rapid identification, annotation and analysis of secondary metabolite biosynthesis gene clusters in bacterial and fungal genome sequences. Nucleic Acids Res.

[40] Ming G, Ying W, Junjun S, Lihua T, Xiaodong S, Jingsong Z, Qi T, Dapeng B (2018). Advances in phylogeny and multi-omics of edible and medicinal fungi. Acta Edulis Fungi.

[41] Guindon S, Dufayard JF, Lefort V, Anisimova M, Hordijk W, Gascuel O (2010). New algorithms and methods to estimate maximum-likelihood phylogenies: assessing the performance of PhyML 3.0. Syst Biol.

[42] Le SQ, Gascuel O (2008). An improved general amino acid replacement matrix. Mol Biol Evol.

[43] Anisimova M, Gil M, Dufayard JF, Dessimoz C, Gascuel O (2011). Survey of branch support methods demonstrates accuracy, power, and robustness of fast likelihood-based approximation schemes. Syst Biol.

[44] Paccanaro A, Casbon JA, Saqi MA (2006). Spectral clustering of protein sequences. Nucleic Acids Res.

[45] De Bie T, Cristianini N, Demuth JP, Hahn MW (2006). CAFE: a computational tool for the study of gene family evolution. Bioinformatics (Oxford, England).

[46] Kim D, Langmead B, Salzberg SL. HISAT: A fast spliced aligner with low memory requirements. Nat Methods. 2015;12(4):357–60. 10.1038/nmeth.3317 PMC4655817 25751142

[47] Huber W. Differential expression analysis for sequence count data. Nat Precedings. 2010;11(R106). 10.1038/npre.2010.4282.1. 10.1186/gb-2010-11-10-r106 PMC3218662 20979621

[48] Young MD, Wakefield MJ, Smyth GK, Oshlack A (2010). Gene ontology analysis for RNA-seq: accounting for selection bias. Genome Biol.

[49] Mao X, Cai T, Olyarchuk JG, Wei L (2005). Automated genome annotation and pathway identification using the KEGG Orthology (KO) as a controlled vocabulary. Bioinformatics (Oxford, England).

[50] Szklarczyk D, Gable AL, Lyon D, Junge A, Wyder S, Huerta-Cepas J, Simonovic M, Doncheva NT, Morris JH, Bork P (2019). STRING v11: protein-protein association networks with increased coverage, supporting functional discovery in genome-wide experimental datasets. Nucleic Acids Res.

[51] Wisniewski JR, Zougman A, Nagaraj N, Mann M (2009). Universal sample preparation method for proteome analysis. Nat Methods.

[52] CM S (1990). Quantitation of protein. Methods Enzymol.

[53] Livak KJ, Schmittgen TD (2002). Analysis of relative gene expression data using real-time quantitative PCR. Methods.

[54] Tyanova S, Temu T, Cox J (2016). The MaxQuant computational platform for mass spectrometry-based shotgun proteomics. Nat Protoc.

[55] MacLean B, Tomazela DM, Shulman N, Chambers M, Finney GL, Frewen B, Kern R, Tabb DL, Liebler DC, MacCoss MJ (2010). Skyline: an open source document editor for creating and analyzing targeted proteomics experiments. Bioinformatics (Oxford, England).

[56] Nur Husna SM, Tan HT, Mohamud R, Dyhl-Polk A, Wong KK (2018). Inhibitors targeting CDK4/6, PARP and PI3K in breast cancer: a review. Ther Adv Med Oncol.

[57] Miller GL (1959). Use if dinitrosalicyclic acid reagent for determination of reducing sugar. Anal Chem.

