# Supplementary figures and images for: Key metabolism pathways and regulatory mechanisms of high polysaccharide yielding in Hericium erinaceus
Source: BMC Genomics. 2021 Mar 6;22:160. doi: 10.1186/s12864-021-07480-x (PMC7937317; doi:10.1186/s12864-021-07480-x)

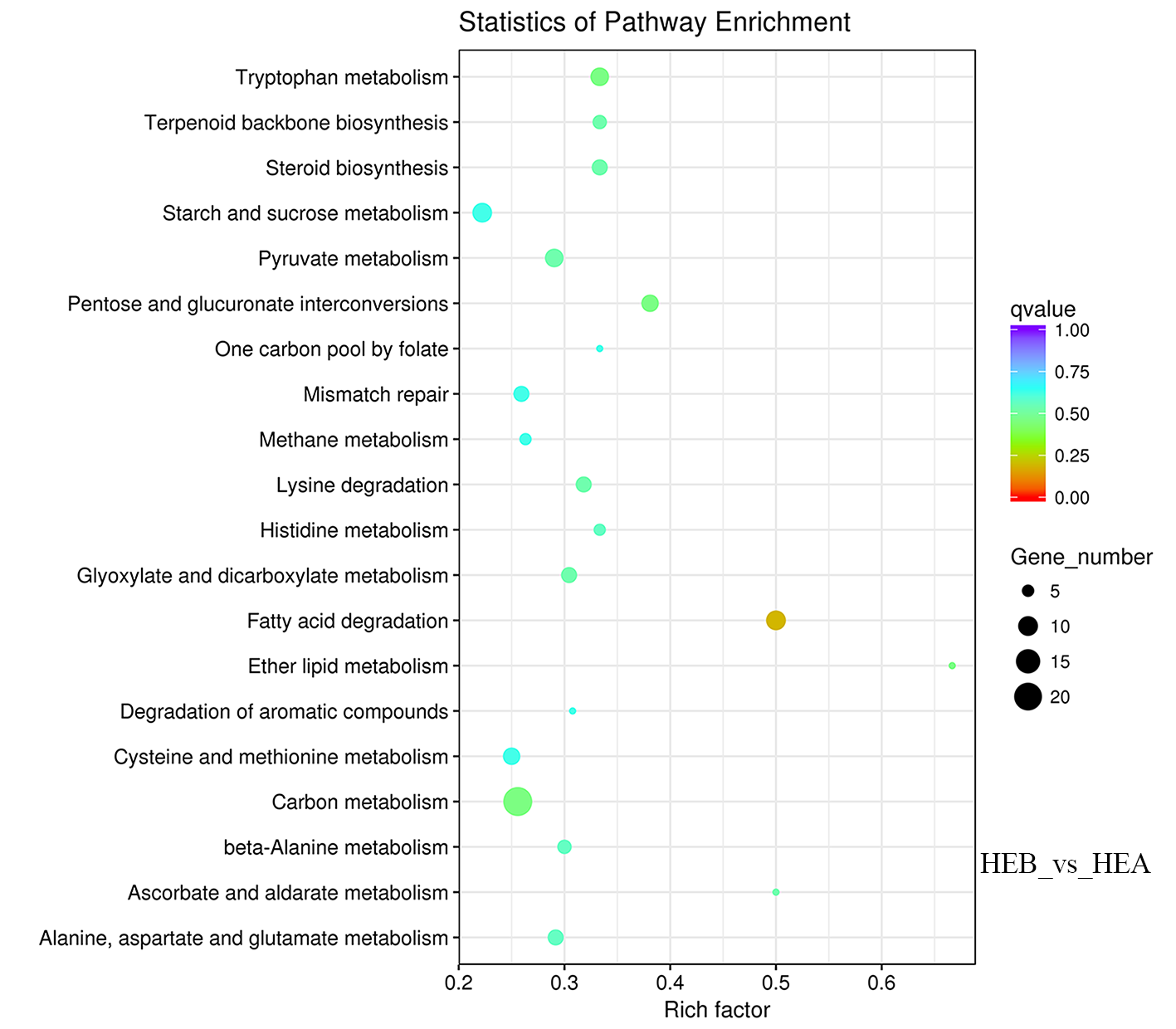

Supplement: Supplementary file 1 — Additional file 1. KEGG pathway enrichment of the significantly upregulated genes in HEB_vs_HEA. [file 12864_2021_7480_MOESM1_ESM.tiff]

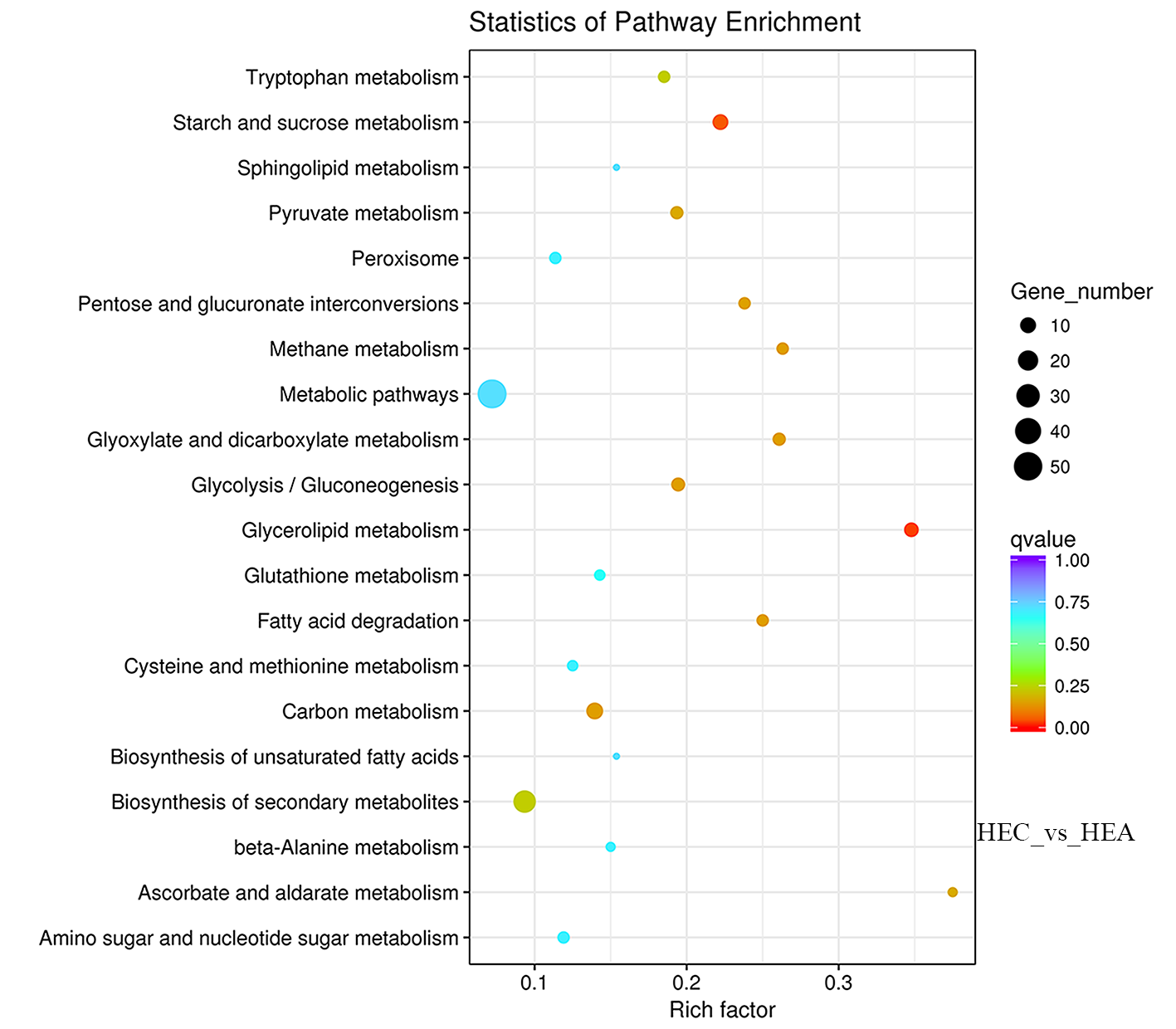

Supplement: Supplementary file 2 — Additional file 2. KEGG pathway enrichment of the significantly upregulated genes in HEC_vs_HEA. [file 12864_2021_7480_MOESM2_ESM.tiff]

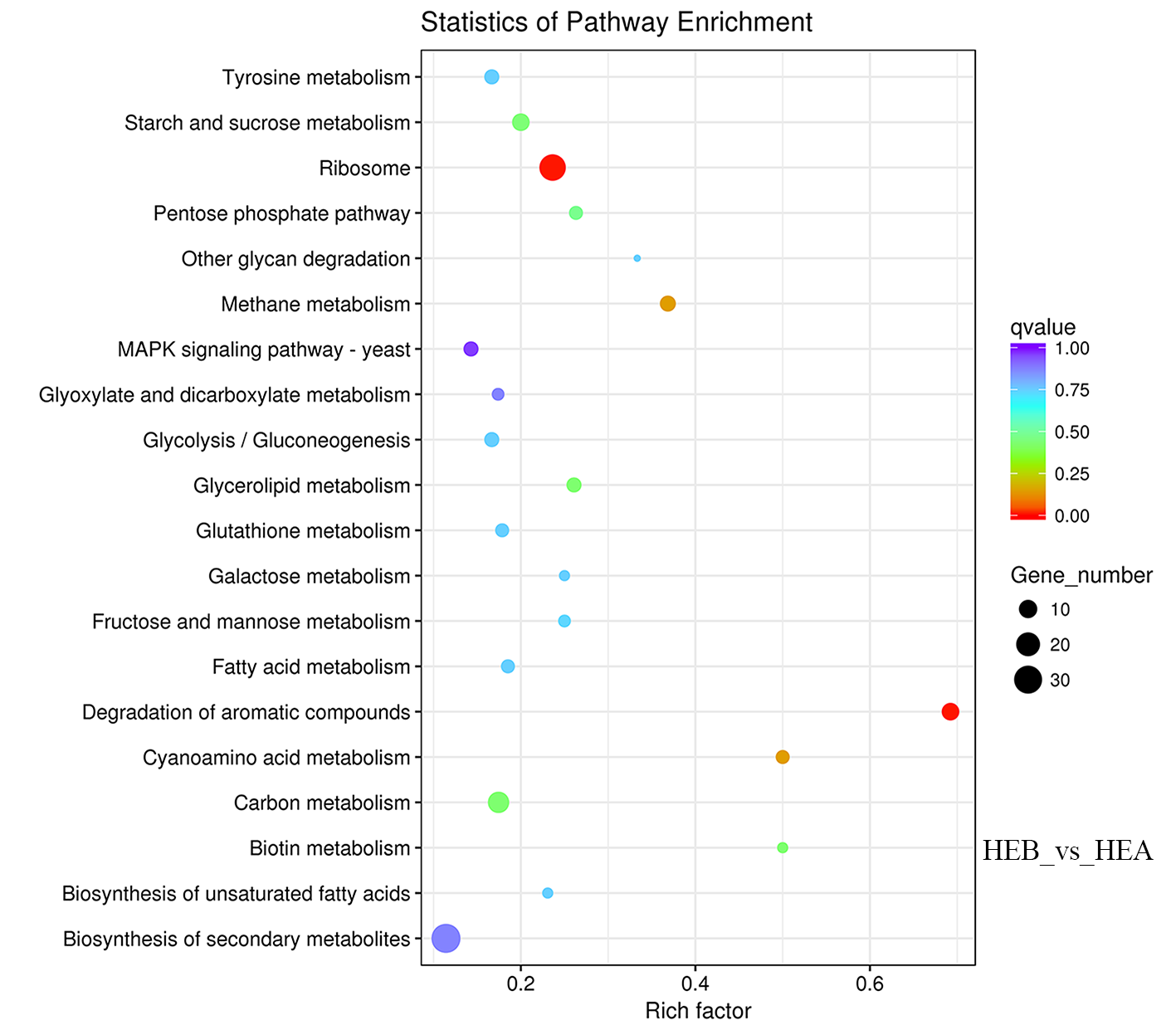

Supplement: Supplementary file 3 — Additional file 3. KEGG pathway enrichment of the significantly downregulated genes in HEB_vs_HEA. [file 12864_2021_7480_MOESM3_ESM.tiff]

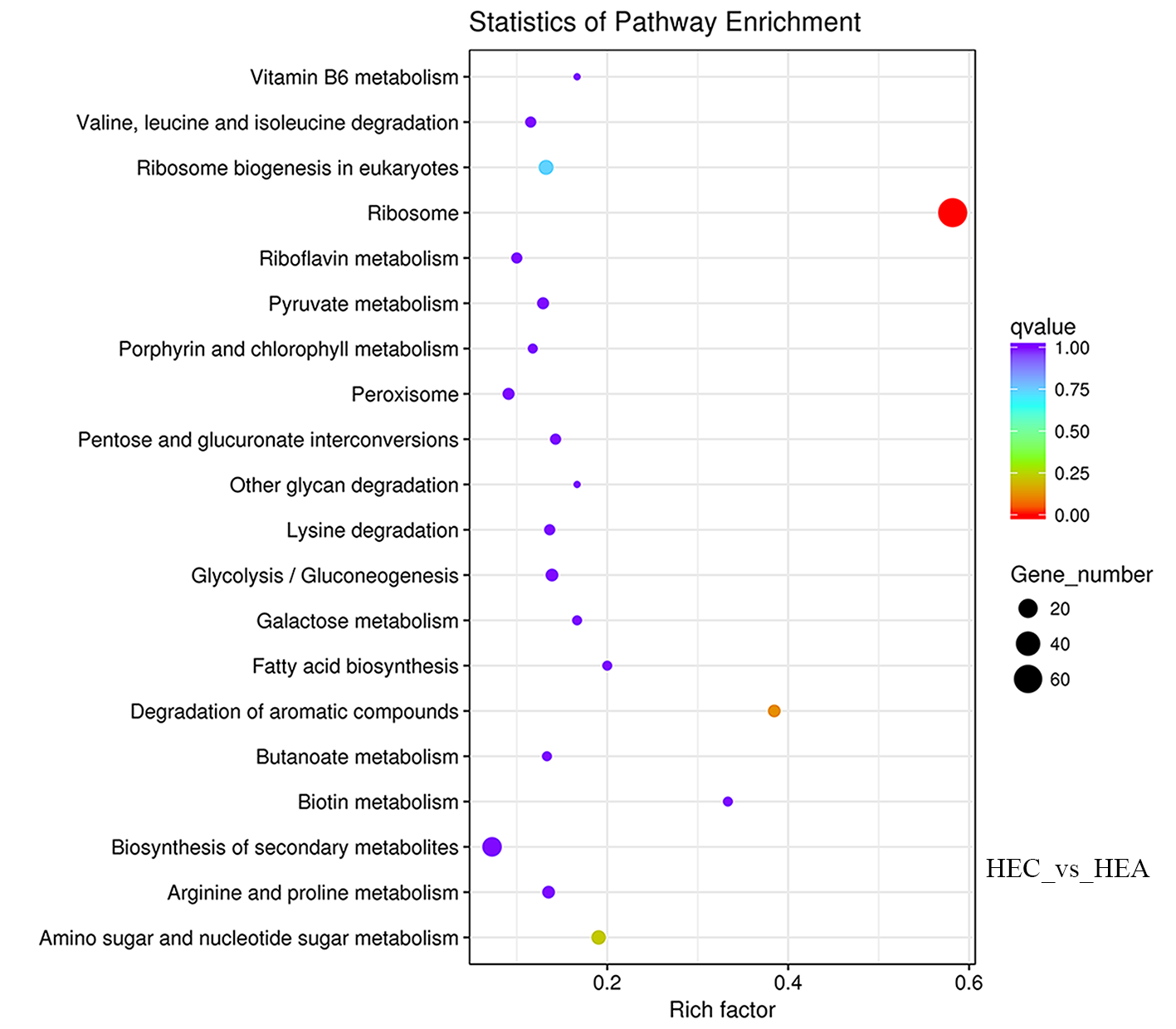

Supplement: Supplementary file 4 — Additional file 4. KEGG pathway enrichment of the significantly downregulated genes in HEC_vs_HEA. [file 12864_2021_7480_MOESM4_ESM.tif]

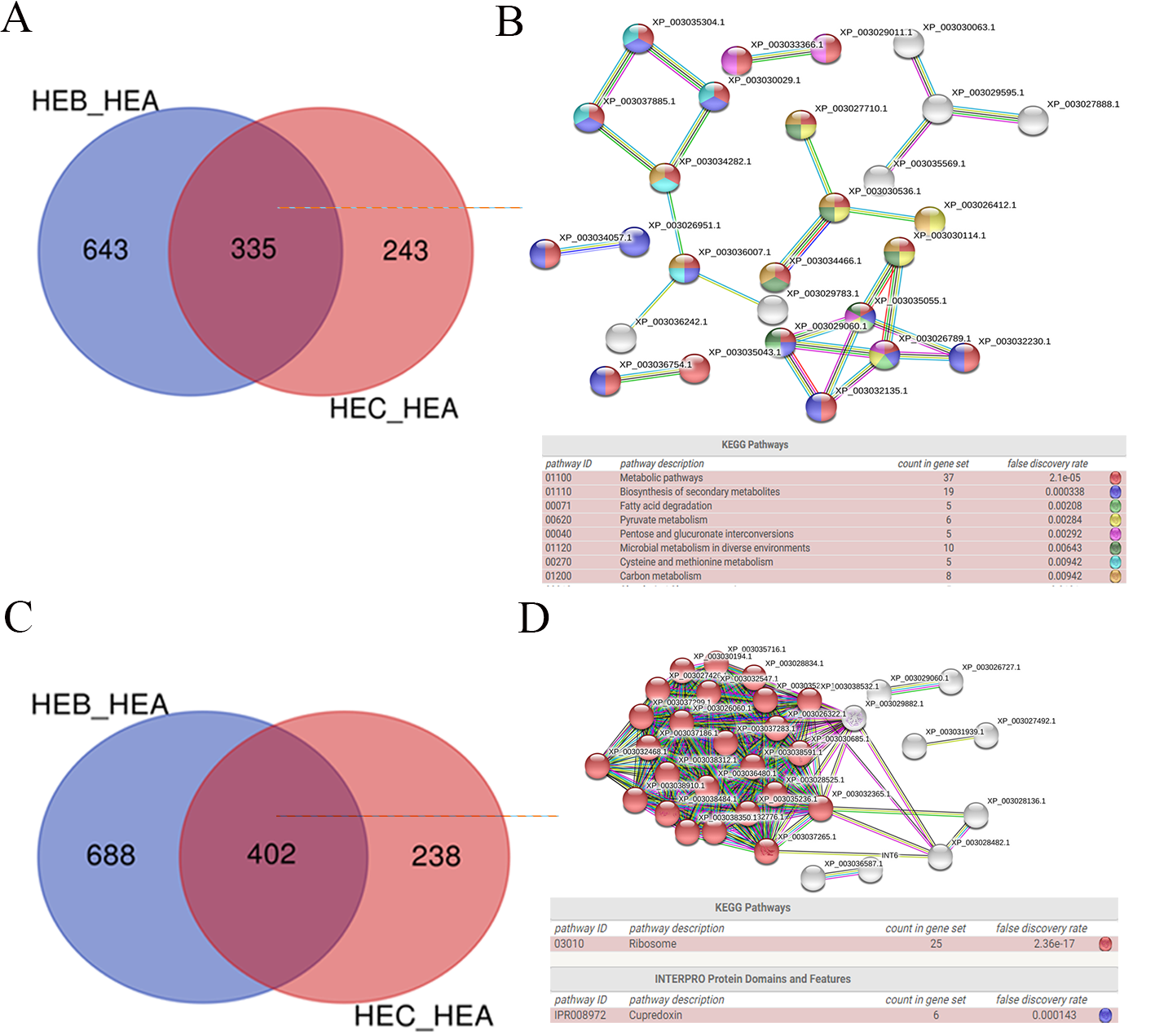

Supplement: Supplementary file 5 — Additional file 5. Transcriptome analysis of H. erinaceus. (A) Venn diagram of significantly upregulated genes from the comparison group of HEC_vs_HEA and HEB_vs_HEA. (B) STRING enrichment analysis of significantly co-upregulated genes. (C) Venn diagram of significantly downregulated genes from the comparison group of HEC_vs_HEA and HEB_vs_HEA. (D) STRING enrichment analysis of significantly co-downregulated genes. [file 12864_2021_7480_MOESM5_ESM.tiff]

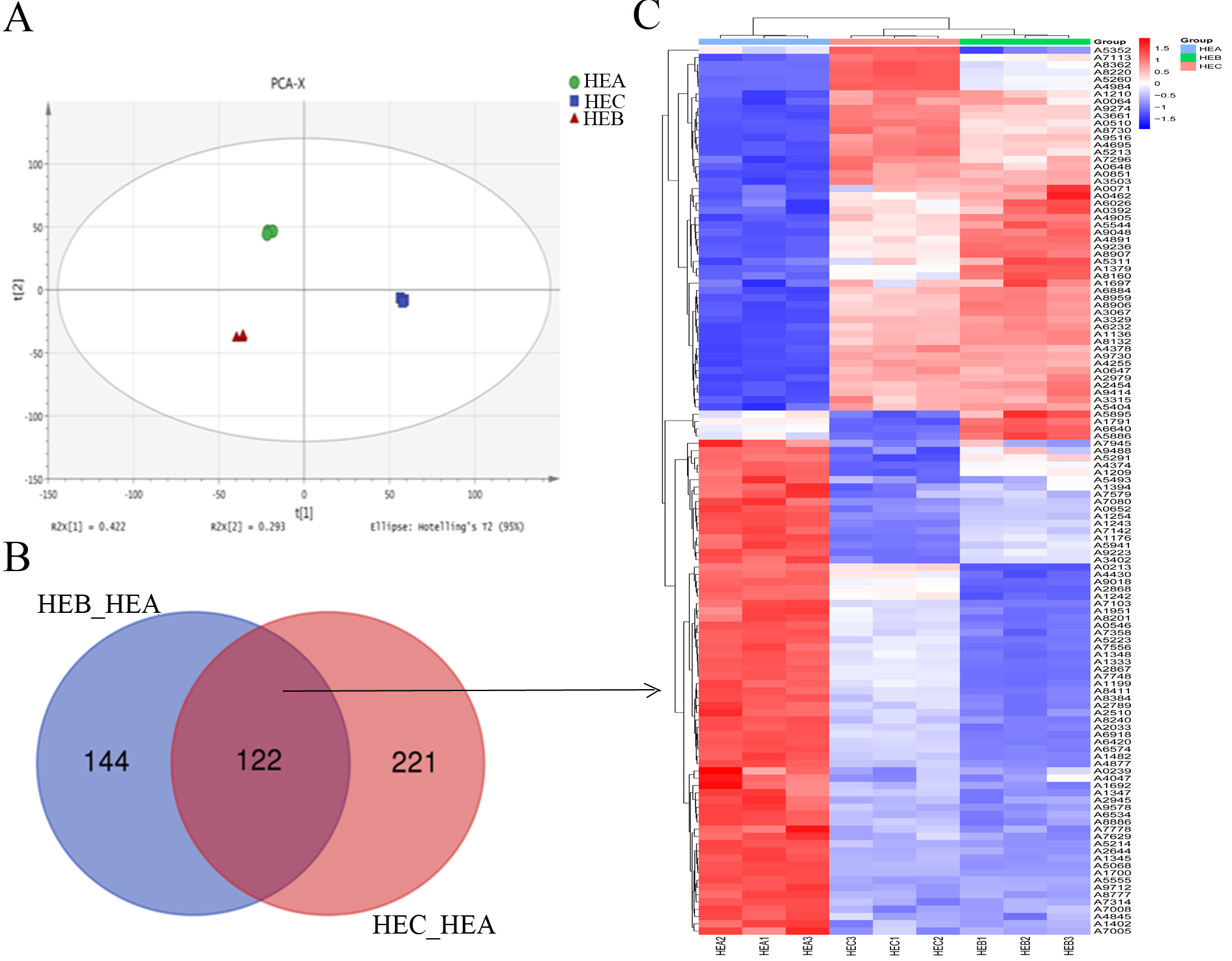

Supplement: Supplementary file 6 — Additional file 6. Proteomics analysis of H. erinaceus. (A) PCA of the expressed proteins. (B) Venn diagram analysis of significantly expressed proteins from the comparison group of HEC_vs_HEA and HEB_vs_HEA. (C) Heatmap clustering of the significantly differentially expressed proteins. [file 12864_2021_7480_MOESM6_ESM.tiff]

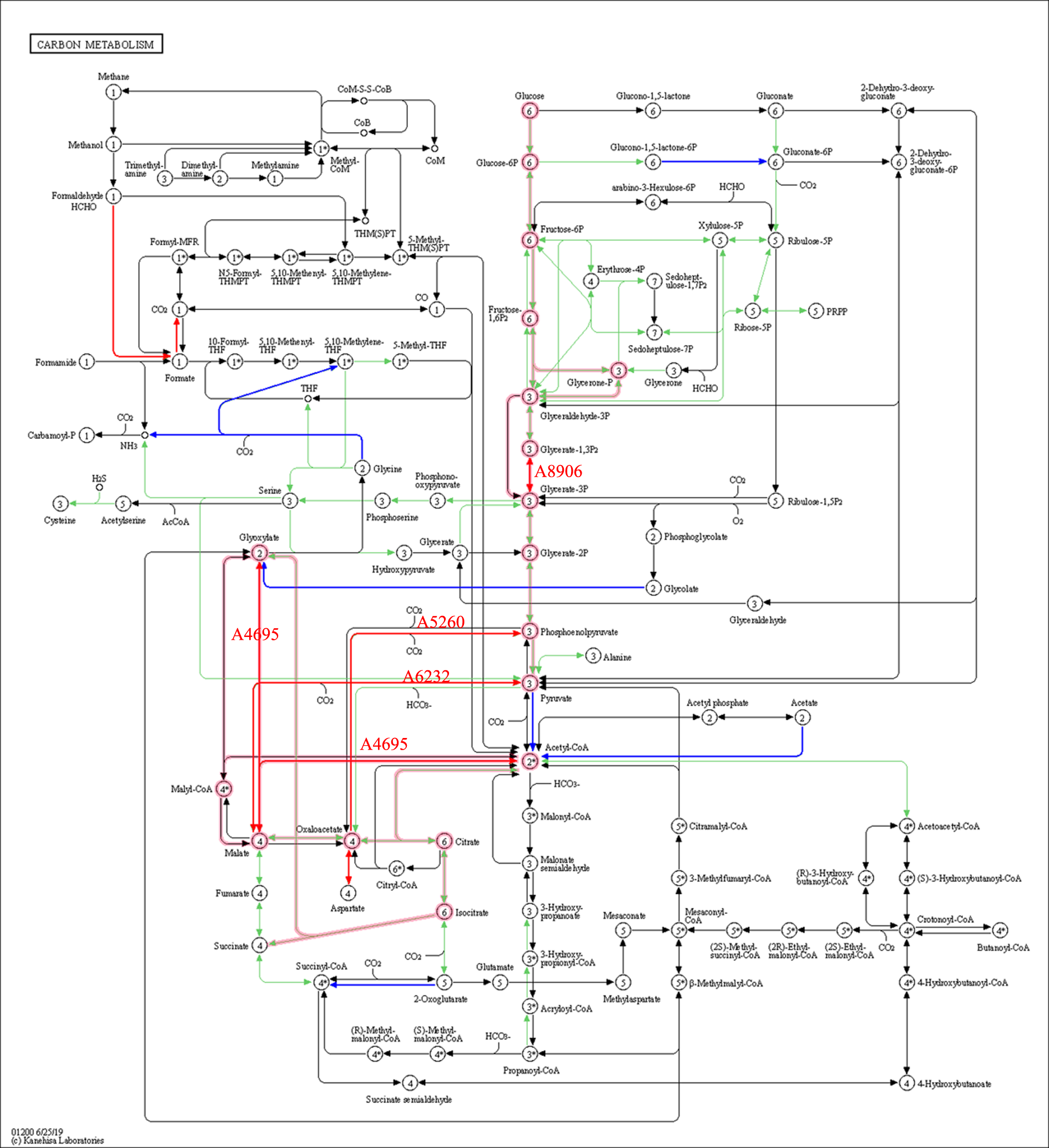

Supplement: Supplementary file 14 — Additional file 14. The KEGG mapping of the enriched pathway modules of carbohydrate metabolism in HEB_vs_HEA. Pink shading lines marked the glyoxylate cycle modules (M00012) and the glycolysis module (M00001). Red lines represent the significantly upregulated proteins. Blue lines represent the significantly downregulated proteins. These images are obtained by KEGG [23]. We have obtained the appropriate copyright permission to modify the KEGG pathways depicted in Additional file 14. [file 12864_2021_7480_MOESM14_ESM.tiff]

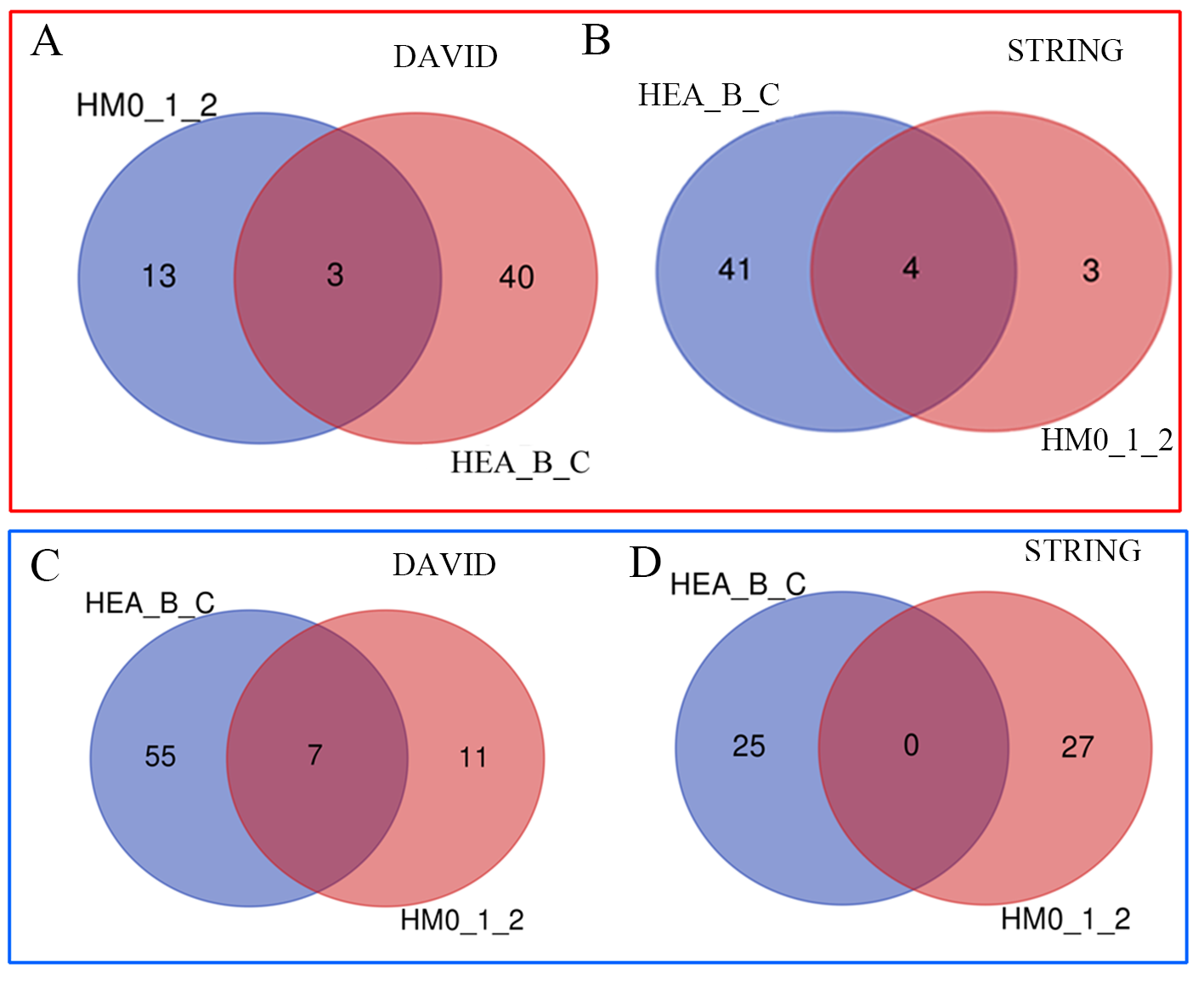

Supplement: Supplementary file 15 — Additional file 15. Venn analysis of the CDC genes in the comparison groups of HEB_vs_HEA and HEC_vs_HEA. (A) Venn analysis of CDC genes based on the DAVID enrichment of the significantly upregulated mRNAs and proteins. (B) Venn analysis of the CDC genes based on the STRING enrichment of the significantly upregulated mRNAs and proteins. (C) Venn analysis of the CDC genes based on the DAVID enrichment of the significantly downregulated mRNAs and proteins. (D) Venn analysis of the CDC genes based on the DAVID enrichment of the significantly downregulated mRNAs and proteins. Note: HM0 = HEA, HM1 = HEC, HM2 = HEB. [file 12864_2021_7480_MOESM15_ESM.tiff]

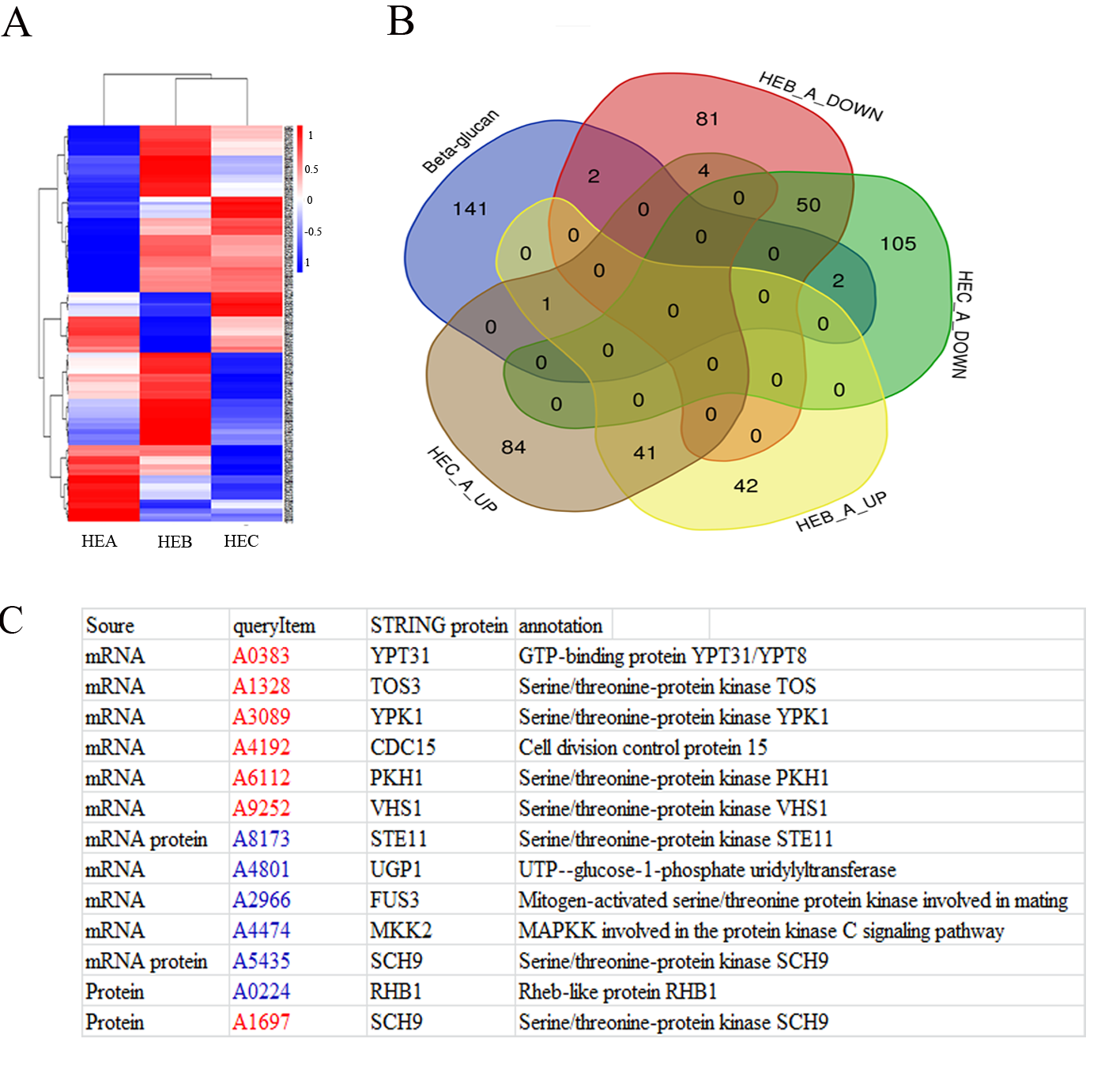

Supplement: Supplementary file 18 — Additional file 18. Multi-omics analysis of the GBP in H. erinaceus. (A) Heatmap analysis of expressed mRNA genes involved in the GBP. Red represents high expression. Blue represents low expression. (B) Venn analysis of the differentially expressed proteins involved in the GBP. (C) The list of DEGs occurred in HEB_vs_HEA and HEC_vs_HEA involved in the GBP. Red represents an apparent upregulation; Blue represents an apparent downregulation. The DEGs were obtained from transcriptome and proteomics, respectively. [file 12864_2021_7480_MOESM18_ESM.tiff]

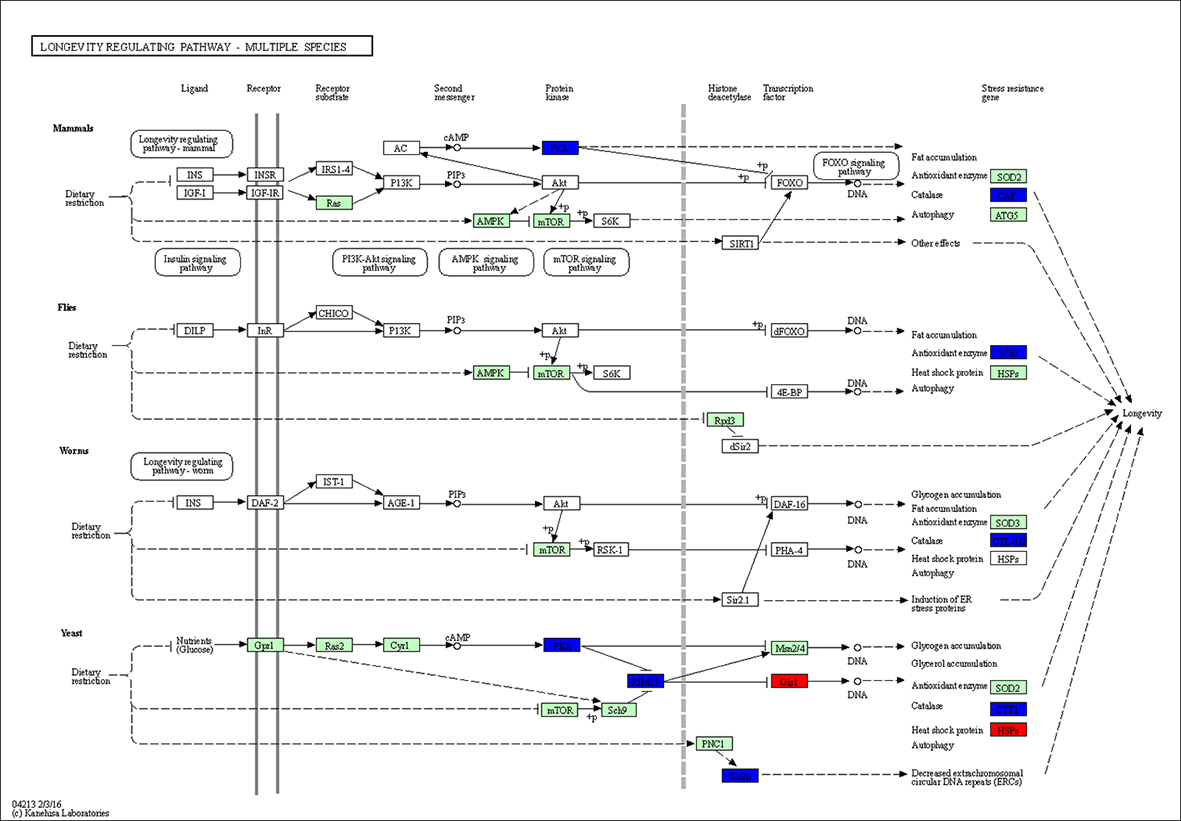

Supplement: Supplementary file 19 — Additional file 19. The KEGG mapping of the enriched pathway of longevity regulation in HEC_vs_HEA. The red box represents the significantly upregulated proteins. The blue box represents the significantly downregulated proteins. These images are obtained by KEGG [23]. We have obtained the appropriate copyright permission to modify the KEGG pathways depicted in Additional file 19. [file 12864_2021_7480_MOESM19_ESM.tiff]

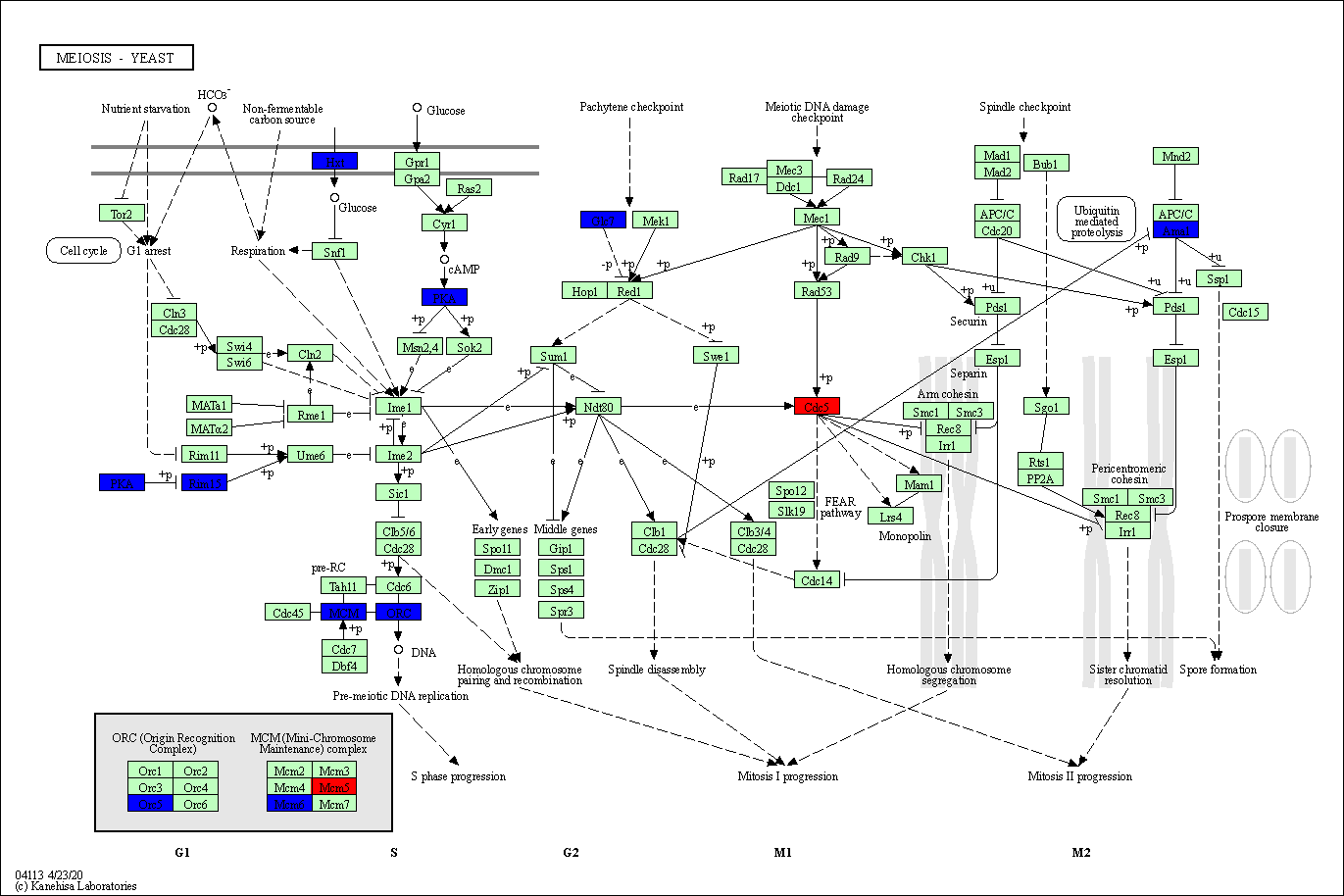

Supplement: Supplementary file 20 — Additional file 20. The KEGG mapping of the enriched pathway of meiosis in HEC_vs_HEA. The red line represents the significantly upregulated proteins. The blue line represents the significantly downregulated proteins. The red box represents the significantly upregulated proteins. The blue box represents the significantly downregulated proteins. These images are obtained by KEGG [23]. We have obtained the appropriate copyright permission to modify the KEGG pathways depicted in Additional file 20. [file 12864_2021_7480_MOESM20_ESM.tiff]
